# Influence of Health Literacy on Effects of Patient Rating Websites: Survey Study Using a Hypothetical Situation and Fictitious Doctors

**DOI:** 10.2196/14134

**Published:** 2020-04-06

**Authors:** Peter Johannes Schulz, Fabia Rothenfluh

**Affiliations:** 1 Università della Svizzera Italiana Lugano Switzerland; 2 Comparis Company Zurich Switzerland

**Keywords:** physician rating websites, warning messages, experiment, physician competence assessment, patient feedback

## Abstract

**Background:**

Physician rating websites (PRWs) are a device people use actively and passively, although their objective capabilities are insufficient when it comes to judging the medical performance and qualification of physicians. PRWs are an innovation born of the potential of the Internet and boosted very much by the longstanding policy of improving and encouraging patient participation in medical decision-making. A mismatch is feared between patient motivations to participate and their capabilities of doing so well. Awareness of such a mismatch might contribute to some skepticism of patient-written physician reviews on PRWs.

**Objective:**

We intend to test whether health literacy is able to dampen the effects that a patient-written review of a physician’s performance might have on physician choice.

**Methods:**

An experiment was conducted within a survey interview. Participants were put into a fictitious decision situation in which they had to choose between two physicians on the basis of their profiles on a PRW. One of the physician profiles contained the experimental stimulus in the form of a friendly and a critical written review. The dependent variable was physician choice. An attitude differential, trust differential, and two measures of health literacy, the newest vital sign as an example of a performance-based measure and eHealth Literacy Scale as an example of a perception-based measure, were tested for roles as intermediary variables. Analysis traced the influence of the review tendency on the dependent variables and a possible moderating effect of health literacy on these influences.

**Results:**

Reviews of a physician’s competence and medical skill affected participant choice of a physician. High health literacy dampened these effects only in the case of the perception-based measure and only for the negative review. Correspondingly, the effect of the review tendency appeared to be stronger for the positive review. Attitudes and trust only affected physician choice when included as covariants, considerably increasing the variance explained by regression models.

**Conclusions:**

Findings sustain physician worries that even one negative PRW review can affect patient choice and damage doctors’ reputations. Hopes that health literacy might raise awareness of the poor basis of physician reviews and ratings given by patients have some foundation.

## Introduction

### Physician Rating Websites

Physician rating websites on the internet (PRWs) came about as a synergy between technological innovation and social reform. The innovation, of course, was the advent and rapid development of the internet. The social reform linked to it was the longstanding public health policy of encouraging patients to participate more actively and autonomously in their health care and preservation.

A key element of the Web 2.0 is electronic world of mouth (eWOM), through which consumers have the chance to evaluate any conceivable product or brand [1] and read evaluations posted by others. This increasingly affects consumer choices [2] and shifts the consumer into a new, more powerful position [3,4]. Some have seen this as generating more equality and democracy in the relationship between seller and customer [5].

eWOM devices soon reached into the field of health care, with PRWs as a prominent example. PRWs are promoted as a medium to increase transparency by communicating health care consumer experiences physicians to a large audience. These websites are becoming increasingly popular; as many as 59% of participants in a representative American study indicated that PRWs were important when choosing a doctor [6], while 25% of Germans [7] have searched for a doctor on the internet. Physician reviews are becoming more and more commonplace, and awareness of PRWs seems to be high in the general population [6,8]. The simple, easily comprehensible, narrative nature of physician rating websites seems to catch users’ attention and appeal to them more than formal quality information such as academic qualifications, degrees, or areas of specialization [9]. Yet, it is largely one-sided communication. Less than 2% of the reviews are responded to by physicians. The share has been growing recently, but is still very low [10].

The benefits of PRWs for health care consumers’ physician choice have been debated [11-13]. Physicians often feel discomfited with open and nonregulated patient feedback platforms [14,15], mainly because they assume health care consumers do not have enough knowledge to pass judgment on a trained physician’s diagnosis and therapeutic recommendations [16].

Assessment of the quality of health care by medical laity differs from expert assessments and is likely to be erroneous, ill-founded, or irrelevant. Decisions based on these assessments, or so health professionals fear, run a high risk of not being in the patient’s best interest as health professionals would see it. In helping the development of PRWs, the ideal of health literacy may have created a form of health communication that is likely to have many drawbacks.

On a more general level, patient satisfaction is not necessarily related to objective outcome-of-care assessments [17-20]. PRW reviews or ratings were found to be only selectively [21,22], weakly [23], or not at all associated to objective measures of quality such as mortality rates [24,25] or surgeon volume. Furthermore, patient judgment of health care quality may be clouded by circumstantial factors. For example, a study by Swiss researchers found in a pre-post test assessment that the renovation of a medical practice led patients to give better ratings not only to the practice infrastructure but also the quality of the staff and the care received, even though personnel and care remained the same [26].

Physicians and patients alike doubt the qualification of patients to pass judgment of the medical qualification of physicians or the technical and outcome aspects of care [27-29]. Patients preferred recommendations from experts when choosing a physician because they perceived them to be more trustworthy and of higher expertise than reviews from patients [30].

As a patient review of a physician’s medical capabilities often is communication from a source whose insight into medical knowledge cannot level with a university-trained physician, the question is raised, does it matter? It will matter if the reviews have effects, for instance, on physician choice. This study investigated the effect of favorable and unfavorable patient reviews of a physician’s medical capabilities on patient attitudes toward a physician and their choice of physician. A second concern was possible ways to dampen these effects, and here health literacy comes into view.

### Patient Participation and Health Literacy

A more active patient participation in health care is generally wished for, for medical reasons—it accelerates, some claim, the process of healing and improves health outcomes [31]—as well as for social values that grant individuals mastery over their own lives, as a well-known definition of empowerment has it [32]. Limited health literacy is found in substantial minorities of populations [33,34] and associated with bad self-care [35-37], bad general health [38,39], and premature death [40]. High health literacy is associated with a number of positive consequences, such as improved disease management [41].

As a young concept, health literacy was restricted to basic understanding of health matters. It was defined as the ability “to obtain, process, and understand basic health information and services needed to make appropriate health decisions” [42]. Later conceptualizations included more demanding abilities in the notion, among them the ability to critically assess the trustworthiness and credibility of the sources of health information [43]. Such ability would make, one can argue, people with high health literacy recognize the questionable nature of the source of physician ratings and consequently activate their skepticism of the credibility of the raters. This should assuage the effects of the reviews.

### Hypotheses

The most fundamental relationship to be considered is the effect of positive and negative PRW text reviews of a physician’s performance. The effect considered foremost is on health care consumers’ choices of a physician. This research interest is captured in the first hypothesis:

- Hypothesis 1: Health care consumers who read a positive text review on the competence of a fictional physician will choose this physician more often than consumers who read a negative text review.

Studies from adjacent fields suggest that negative information is especially powerful in impacting internet users’ opinion on a given subject. Specifically, negative information catches people’s attention more than positive content as it refers to hazards, the warning against which was crucial for human survival from an evolutionary perspective [44]. A stronger effect of negative reviews was also found in eWOM for product choices [45] as well as credibility [46,47]. Some medical research, however, finds an equal effect on PRW users’ choices of positive and negative reviews. An American study, for instance, found that 37% of PRW users avoided a physician when they were confronted with bad reviews, while 35% decided to consult a physician when facing positive evaluations [6,7,48-50]. These inconclusive findings suggest a research question on the influence of review valence on the strength of the effect of negative and positive reviews.

- Research Question 1: Do negative or positive text reviews have a stronger effect on patient choice of a physician?

The analysis must be mindful of the fact that PRW reviews are predominantly positive (ranging from 63% to 88%) as studies from websites in the United States [51-54], Germany [55], the United Kingdom [56], Poland [57], and China [58] report.

People with high health literacy acknowledge that a lay person’s ability to assess a physician’s medical competence is limited and do not consider reviews when they make their physician choice. Technically speaking, this means high health literacy would diminish the relationship between review tendency and physician choice and attitudes. In other words: health literacy would negatively moderate the effect of the review. This is the major relationship we want to demonstrate in this research (Hypothesis 2).

- Hypothesis 2: High health literacy will weaken the effect of review tendency on attitudes and physician choice (negative moderation effect).

The association between review tendency and behavioral intentions, physician choice in our case, might be made more complex by attitudes or trust. Several models are conceivable. Attitudes and trust may mediate the effect of review tendency on physician choice, and attitudes and trust might interfere in the assuaging role of health literacy if there is such.

- Research Question 2: How do attitudes and trust affect the influences on patient choice of a physician?

## Methods

### Data Collection and Sample Characteristics

An online survey was conducted in March 2017 via a contracting firm (Qualtrics) specialized in survey administration and market research data collection. The ethics committee at the Università della Svizzera Italiana confirmed the study was outside the committee’s jurisdiction (CE 2017-1). For inclusion in the study, participants had to be (1) aged 18 years or older, (2) residing in the German part of Switzerland, and (3) fluent in German. Data collection was anonymous, and participants could only participate once. Based on the ultimate of three pretests (n=24 took part in total), the median survey completion time was 14 minutes. To ensure validity, participants who used less than two-thirds of the median time (9 minutes) were excluded. Participants who did not pass the manipulation check, in which they were asked about the number of text reviews present on each of the two physician profiles, were screened out as well. A total of 258 participants passed the time and manipulation checks. When the data were screened for automatic response behavior, 4 more cases were excluded, yielding a final sample of 254 cases. The sample was equally split in terms of gender (129/254, 50.8% female), had education levels that are comparable to the Swiss general population [59], and participants were on average 47.8 (SD 16.05) years old (range 18-85 years; see Table 1).

**Table 1:** Sociodemographic characteristics of participants by experimental group (n=254).

Characteristic		Positive review n=126	Negative review n=128	*P* value
**Gender, n (%)**				**.71^a^**
	Male	59 (46.8)	66 (51.6)	—
	Female	67 (53.2)	62 (48.4)	—
**Education level, n (%)**				**.32^a^**
	Low (primary or secondary school or apprenticeship)	65 (51.6)	61 (47.7)	—
	Medium (high school or similar)	31 (24.6)	24 (18.8)	—
	High (applied science or university degree)	30 (23.8)	43 (33.6)	—
Age in years, mean (SD)		45.75 (14.46)	51.66 (15.85)	.09^b^

^a^Chi-square test.

^b^One-way analysis of variance.

### Procedures

In the first part of the survey, participants were asked about their search behaviors on the internet and basic sociodemographic questions (Table 1). Next, they were exposed to a hypothetical scenario to introduce them to a physician selection task. The scenario was developed in cooperation with a medical doctor to ensure its reasonability. Specifically, participants were asked to imagine to have been hurt by a nail during their move to a new town. Subsequently, the wound got infected. Because they had not met anyone yet to ask for a recommendation of a physician, they would look for one online and find the PRWs of two doctors, among whom they would need to choose one. The complete instructions can be found in Multimedia Appendix 1. The PRWs were designed as to resemble real PRWs as much as possible.

**Figure 1:**
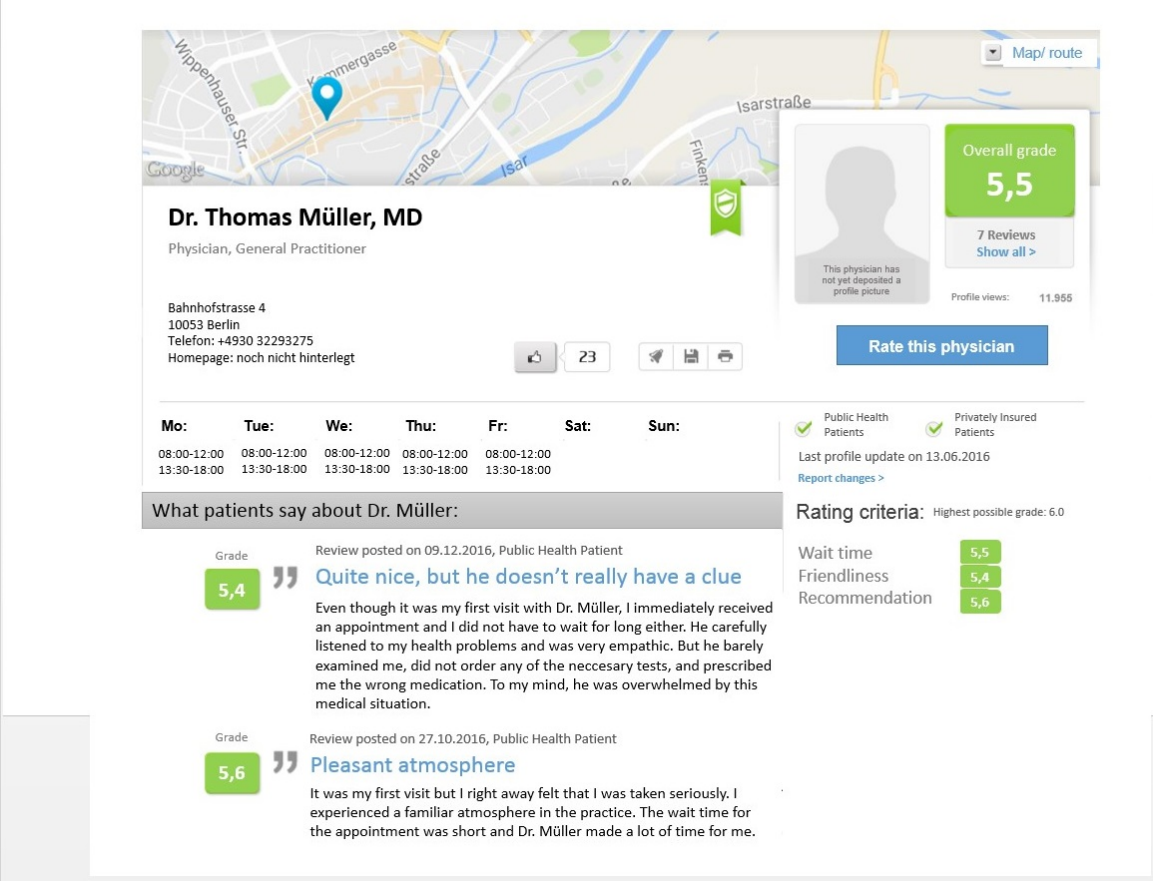
Stimulus material for unfavorable review condition.

The fictional doctors were named Dr med Thomas Müller and Dr med Michael Schmidt, both common German names to provide a realistic scenario. Based on the finding that users pay more attention to written than numerical reviews [9], one of two written reviews in Dr Müller’s profile was manipulated (assessing his competence positively or negatively), and participants were randomly assigned to either one of two conditions. Recruitment and randomization to experimental and control conditions followed Qualtrics procedures based on random numbers. As research finds the number of written reviews on PRWs rather small [60,61], we offered two of them on Dr Müller’s rating site, one of which was manipulated, and none on Dr Schmidt’s profile. The numerical scores on both PRWs varied slightly but produced the same average assessment. Dr Schmidt’s PRW was not manipulated and, borrowing an idea from Li and colleagues [62], added solely for the purpose of creating a decision situation (see Figure 1 for an example of the profiles). All relevant information on the website (eg, overall numerical rating score, number of numeric reviews) except the manipulated text review were similar across conditions. We chose the reviews rather than the ratings for manipulation because they are more variable, which will be helpful in future studies. The two profiles differed only minimally in terms of information content to make the choice situation more realistic (eg, house number of the practice address, phone number). The display order of the two doctors’ profiles was randomized to account for primacy effects. There were actually more differences as the experiment was in a 2×2 factorial design with the presence or absence of a warning message as second independent variable, but this is of no relevance to the analyses reported in this article and is therefore not mentioned further.

### Dependent and Intervening Measures

For physician choice, the major dependent variable in all analyses, participants were asked which one of the two physicians they would choose (7=definitely Dr Müller, 1=definitely Dr Schmidt). As only Dr Müller had received reviews, the scale values are referred to as preference for Dr Müller. The attitudinal variables pertaining to RQ2 included an attitude differential and a trust differential. Attitude toward each of the profiles was surveyed based on semantic differential 5-point scales consisting of 8 items. Adjective pairs included, for example, helpful versus useless, realistic versus forged, reliable versus unreliable. One item was reversed to check users’ attention to account for automatic response behavior. The average score of the 8 items was calculated and the difference between the two physicians calculated and used in the analysis. This was done as Dr Schmidt’s evaluation was of no interest, while the comparison between the two doctors was.

Trust in the respective physician, specifically the impression participants formed of him, was measured by three items: Dr X makes a good impression on me, Dr X convinces me, I would trust Dr X (1=not at all true, 7=entirely true). A cumulative score was calculated and subsequently averaged after reliability checks were found to be satisfactory. All scales achieved satisfactory internal reliability with Cronbach alpha above .85. As was done for attitudes, the variable used was the difference between the two physicians’ cumulative scores. An overview of the variables and scale details can be found in Table 2, and the complete questionnaire in Multimedia Appendix 2.

For the measurement of health literacy, two types of indicators are available. The classic measures are based on the performance of patients, as in their ability to correctly pronounce medical terms (Rapid Estimate of Adult Literacy in Medicine) [63] or complete omissions in sentences describing medical matters (Short Test of Functional Health Literacy in Adults) [64]. As the concept was extended by more demanding cognitive abilities, new measures were introduced, based on the (self)perception of health care consumers. While performance-based measures can claim a high degree of objectivity, perception-based measures are undoubtedly subjective and therefore prone to be affected by a person’s biases, ideals, and needs. We chose two measures, one of each type: the eHealth Literacy Scale (eHEALS) measure [65], in a validated German translation [66], as an example of the perception-based type and the newest vital sign (NVS [67]), also in the German version, for the performance-based measure.

The original eHEALS consists of 8 items; we used 7 of them, probably not wisely, because the item “I know what health resources are available on the internet” did not work well in some of our studies, but did in others [68]. Users rated the items on 5-point scales (1=strongly agree, 5=strongly disagree); the items were averaged and had a satisfactory reliability (Cronbach alpha=.86).

The NVS consisted of items assessing reading comprehension and calculation questions referring to the information given on a fictitious ice cream nutrition label. For every correct answer, one point was assigned, adding up to a final score between 0 and 6, with higher scores indicating higher literacy levels. Applying the standard procedure for translating such materials, the German version was translated from English independently by two native speakers of German and then backtranslated independently by two native speakers of English, discussing and resolving discrepancies at both stages. A separate formal validation was not conducted. For an overview of the scales, see Table 2.

**Table 2:** Independent and moderator variables, their function and descriptive statistics, scale, and item properties (n=254).

Variable	Function in analysis	Items n	Scale range	Mean (SD)	Reliability Cronbach alpha
Performance-based health literacy (NVS^a^)	Moderator in analysis pertaining to H2^b^	6	0 to 6	4.29 (1.72)	n/a
Perception-based health literacy (eHEALS^c^)	Moderator in analysis pertaining to H2	7	1 to 5	3.65 (0.68)	.86
Physician choice (7=Dr Müller, 1=Dr Schmidt)	Dependent variable	1	1 to 7	3.48 (1.71)	n/a
Attitude differential Dr Müller over Dr Schmidt	Intermediary	8	–4 to 4	0.29 (0.79)	.89
Trust differential Dr Müller over Dr Schmidt	Intermediary	3	–6 to 6	0.29 (1.68)	.88
Skepticism of eWOM^d,e^	Not in hypotheses but helps explain findings	10	1 to 5	2.96 (0.69)	.88
Usefulness of PRW^f^ information^g^	Not in hypotheses but helps explain findings	3	1 to 7	4.10 (1.59)	.95

^a^NVS: Newest Vital Sign.

^b^H2: hypothesis 2.

^c^eHEALS: eHealth Literacy Scale.

^d^eWOM: electronic word of mouth.

^e^Adapted from Grabner-Kräuter and Waiguny [69].

^f^PRW: physician rating website.

^g^Adapted from Diviani et al [68].

Table 2 lists two variables not entered in the hypotheses and research questions but later used for interpretation of results. The perceived usefulness of reviews on websites was assessed (1=not at all, 7=very useful) based on 3 items adapted from Grabner-Kräuter and Waiguny [69] (see Multimedia Appendix 2 for questionnaire). A cumulative average score was calculated after the scale was checked for internal reliability.

Skepticism toward eWOM adapted to the PRW context [70] was assessed on a 5-point scale (1=completely disagree, 5=completely agree) applied to 8 items inquiring about participants’ agreement with statements. Individual item scores were also added up and averaged after internal reliability checks were found to be satisfactory.

All variables were coded in a direction so that correlations between hypothesized or intuitively associated variables were positive. In particular, high scale values indicate a friendly review tendency, a stronger preference for Dr Müller over Dr Schmidt, high level of health literacy (on both measures), more favorable attitudes toward Dr Müller compared with Dr Schmidt (attitude differential), and higher trust in Dr Müller compared with Dr Schmidt (trust differential). The hypothesized negative moderator role of health literacy on the association between review tendency and preference for Dr Müller would show in negative effect coefficients.

### Analysis

The collected data were analyzed quantitatively using SPSS Statistics 23.0 software package (IBM Corp). First, data were analyzed for uni- and multivariate outliers, nonnormality, and missing data [71]. H1 was tested and RQ1 answered by comparing means, and *t* tests, chi-square difference tests, and one-way analysis of variance (ANOVA) were applied to assess potential differences between the experimental groups. To test the moderating effect of health literacy on choice behavior (H2), moderation analyses (model 1) based on the PROCESS macro for SPSS version 2.16.3 (AF Hayes) were applied [72]. For the analysis of the final model, which includes attitudes and trust as covariates (RQ2), we added these covariates to the PROCESS command line in model 1, the cov option).

## Results

### Effect of Review Tendency on Physician Choice and Attitudes

H1 held that health care consumers who encountered a positive text review of Dr Müller’s competence would choose him more often than consumers who read a negative review. Individuals who read a positive assessment of Dr Müller’s competence were more willing to choose him than participants who saw the negative assessment. ANOVA for testing significance found an impact of the review tendency on physician choice, with a significant main effect. The average scores and details of significance tests are shown in Table 3. These findings indicate that the tendency of reviews of a physician’s competence impact users’ choices. H1 is supported: reviews matter.

The group confronted with a negative review of Dr Müller rated their attitudes toward the two physicians similarly. When there was a positive review of Dr Müller, his mean attitudinal rating was clearly higher than Dr Schmidt’s. Trust in Dr Müller exceeded trust in Dr Schmidt by 1.02 (SD 1.49) scale points when Dr Müller was reviewed positively and fell short by –0.43 scale points when the rating was negative. Both differences were highly significant (attitudinal differential *F*_1,252_=41.151, *P*<.001; trust differential *F*_1,252_=134.656, *P*<.001). These results indicate that, aside from physician choice, health care consumers’ attitudes and trust can also be affected by review tendency. For a simple overview of the difference review tendency makes, see Table 3.

RQ1, on the magnitude of effect of negative and positive reviews, can be tentatively answered in favor of positive reviews. The physician choice measure ranged from 1 to 7 and has 4 as the meaningful middle point, which indicates similar likelihood to choose either one of the doctors. In the condition of a negative review, the score fell 0.31 scale points down from the middle on the preference for Dr Schmidt’s side, while with a positive review it was 1.37 points on the preference for Dr Müller’s side. As just shown, attitude and trust differentials as indicators of the evaluation of the two physicians reacted significantly more strongly to positive than negative reviews (0.58 vs 0.01 in case of the attitude differential, and 1.02 vs –0.43 in case of the trust differential). A similar test for the more obvious outcome variable, physician preference, is not possible, as the difference was asked in just one question and the deviation from the scale mean is good for illustration but is no basis for significance testing. Without a strict testing for significance, these results suggest rather than validly support that in our setting the positive review seemed to have had the stronger effect on preference, too.

**Table 3:** Preference and assessment of fictitious doctors in different conditions.

Experimental condition		Line #	Prefer Dr Muller over Dr Schmidt	Objects and attitudes of trust	Attitudes	Trust
Negative review on Müller profile		1	3.69 (1.63)	Schmidt	3.18 (0.53)	3.96 (1.08)
Negative review on Müller profile		2	—	Müller	3.17 (0.64)	3.53 (1.22)
Negative review on Müller profile		3	—	Δ (2–1)^a^	–0.01 (0.75)	–0.43 (1.56)
Positive review on Müller profile		4	5.37 (1.34)	Schmidt	3.15 (0.62)	3.91 (1.07)
Positive review on Müller profile		5	—	Müller	3.73 (0.64)	4.94 (1.17)
Positive review on Müller profile		6	—	Δ (5–4)^b^	0.58 (0.72)	1.02 (1.47)
Grand difference as indicator of difference between the conditions Δ (4–1)^c,d^		7	1.68	Δ (6–3)^e^	0.59	1.45
**Significance**		n/a^f^	—	—	—	—
	F/t^g^	—	80.55	—	6.415	6415
	*df* ^h^	—	1250	—	252	252
	*P* value	—	<.001	—	<.001	<.001
	*η*_p_^i^	—	0.244	—	—	—
	*R* ^2^ ^i^	—	.235	—	—	—

^a^Difference between line 2 and line 1 values for attitudes and trust.

^b^Difference between line 5 and line 4 values for attitudes and trust.

^c^Difference between line 4 and line 1 values for prefer Dr Muller over Dr Schmidt.

^d^Positive values indicate higher preference for Dr Müller, more confidence in own decision, and higher trust and better attitudes when the Müller review was friendly.

^e^Difference between line 6 and line 3 values for attitudes and trust.

^f^Not applicable.

^f^F/t: indicator of model fit.

^g^*df*: degree of freedom.

^h^*η*_p_: indicator of association between dependent and independent variables.

^i^*R*^2^: indicator of contribution of independent on dependent variables.

### Adding Health Literacy to the Picture

Our question was whether the effect of review tendency on doctors’ choice was dependent on the level of health literacy. The logic of the moderation analysis is to calculate and assess the impact of the interaction of the independent and moderator variables on top of the main effect of both. We can only speak of moderation if the interaction can be shown to exert a significant influence beyond the main effect of review tendency as the hypothesized independent variable and health literacy as the hypothesized moderator. Effects were only found for the eHEALS. Therefore, the analysis proceeds with this measure as indicator of health literacy. The hypothesis was that the more literate the consumer was, the weaker the effect would be of review tendency on the preference for the reviewed doctor. The reason for that is that highly literate people (particularly those who consider themselves as being more familiar with the internet) are less willing to believe in the face value of reviews of doctors as they are posted online. There were two ways to approach this question: (1) use regression models to identify a moderating role of health literacy on the effect of review tendency on preference for the reviewed doctor (Table 4) or (2) analyze the two experimental groups separately.

Regression model 1 shows the effect of the experimental condition on the preference for Dr Müller. When the presence of a negative review was coded 0 and a positive review 1, the constant corresponds to the average of the preference variable under the negative review condition and X is the difference of preference under the positive review condition. This is another way to demonstrate the support that H1 received in our data.

Model 2 adds an influence of health literacy on preference for Dr Müller. This was included not for testing a substantial hypothesis but merely as a built-up to model 3. Unexpectedly, however, model 2 revealed a significance of the association between health literacy and preference. Irrespective of the review tendency, people with a higher health literacy showed a higher preference for Dr Müller over Dr Schmidt. The correlation was *r*=.104; it failed to reach significance in bivariate analysis (*P*=.10) other than in model 2.

Model 3 adds an interaction term to test for the dampening influence of health literacy as formulated in H2. The effect was there, as expected with a negative sign. Model 4 added attitudes, trust, and gender as covariates, which almost doubled the *R*^2^ value from 26.6% to 52.4%. Other models were tested that conceptualized attitude and trust in various mediator and moderator roles. None of these showed any significant contribution of attitudes, trust, and sociodemographic variables.

Technically speaking, as eHEALS health literacy increases by one unit, the difference in preference for Dr Müller according to model 3 between those exposed to a positive review and those exposed to a negative review decreases by –.546. So to say, the moderator quantifies a difference between differences. Other moderation analyses based on the PROCESS macro (model 1) and addressing the influence of attitudes and trust were applied, as well as some according to model 2 with more than one moderator. These analyses did not show any significant moderating effects (RQ2) [72].

The separate analysis of the two experimental groups (positive vs negative review) can be summarized as follows. For those who were exposed to a positive review, preference for the reviewed doctor did not depend on their health literacy levels; whether they considered their health literacy high or low, the share among them who preferred the applauded doctor remained the same. This changed with respect to the participants who were exposed to negative reviews: the lower their literacy, the lower the preference for the criticized doctor would be. In different perspective: the higher the health literacy levels participants showed, the less they were influenced by the negative review to disregard the doctor. As a matter of fact, people with high literacy levels exposed to negative reviews came closer in their choice to those who had seen a positive review of the doctor.

A simple way to illustrate the difference is correlation analysis. For the group that saw a positive review, the correlation between health literacy and preference for Dr Müller was nonexistent (*r*=.01, *P*=.91), while the two variables were associated in the group that saw the negative review (*r*=.228, *P*<.01).

**Table 4:** Various regression models estimating preference for Dr Müller over Dr Schmidt.

Independent variables		Coefficient	Standard error	*t*	*P* value
**Model 1 (*R*^2^=.243)**					
	Constant	3.687	0.132	27.914	<.001
	Review tendency (X)	1.686	0.188	8.986	<.001
**Model 2 (*R*^2^=.255)**					
	Constant	2.675	0.520	5.143	<.001
	Review tendency (X_1_)	1.690	0.186	9.061	<.001
	eHEALS^a^ (X_2_)	0.277	0.138	2.012	.045
**Model 3 (*R*^2^=.266)**					
	Constant	1.621	0.740	2.189	<.05
	Review tendency (X)	3.679	1.017	3.616	<.001
	eHEALS (M)	0.566	0.199	2.836	<.001
	X*M	–0.546	0.274	–1.989	.048
**Model 4 (final model; *R*^2^=.524)**					
	Constant	2.151	0.650	3.309	<.001
	Review tendency (X)	2.384	0.838	2.846	<.01
	eHEALS (M)	0.322	0.165	1.952	.05
	X*M	–0.428	0.225	–1.906	.06
	Covariate trust	0.346	0.063	5.493	<.001
	Covariate attitudes	0.515	0.134	3.846	<.001
	Covariate sex	0.358	0.154	2.318	<.05

^a^eHEALS: eHealth Literacy Scale.

## Discussion

### Principal Findings

Our study showed that when a physician has a patient-written review on their online profile that describes their technical skill and competence negatively, internet users are less willing to choose that doctor and hold more critical attitudes about and trust the doctor less. The opposite is true when reviews are positive. These findings underscore the results from previous studies, which reported that PRW users rely heavily on text reviews when selecting a doctor [1], and confirm that review tendency impacts their perception of the review content [62].

The manipulated review came along with a neutral second one that was not manipulated. The experimental stimulus was inconspicuously placed on the manipulated profile, and the quantitative information on the profile was untouched. Hence, the effects found should be judged considerable. This confirms physicians’ worries about the impact of even one negative, potentially unjustified review [16,73]. As a consequence, the need for better physician protection mechanisms against fake or unjustified reviews remains a priority. For example, if reviews go online only when a quorum of them is available, say 10, the weight of a single negative one will diminish and a more realistic picture of a physician’s true competence [8,73] will emerge. Despite awareness of such balancing efforts, a recent content analysis on PRWs found that only a small minority of PRWs demand a quorum before reviews go online [74], and many physicians in the country have far fewer than 10 reviews on their websites [60].

On the other hand, our data and analyses do not show the slightest hint of halo effects. That is to say, attitudes toward Dr Schmidt were not affected by the reviews Dr Müller received, neither critical nor friendly. Such halo effects are not inconceivable, and they make health care consumer reactions even more unpredictable. Their absence, moreover, does not mean that a physician whose performance was not reviewed remains unaffected. The effect is on patient behavior that leads to increased or deteriorating demand for the services a physician offers.

The stronger effect of positive compared with negative reviews rests on the assumption that Dr Müller without review would receive judgments similar to Dr Schmidt without review. Given that the profiles are virtually identical, this assumption is reasonable, but we cannot be 100% sure as there were no profiles of Dr Müller without reviews. Data even show a slightly better liking of Dr Müller (MMüller 3.45, MSchmidt 3.16, *t*_253_=5.755, *P*<.001 in paired samples *t* test) and somewhat more trust put in him across the total sample (MMüller 4.23, MSchmidt 3.94, *t*_253_=5.755, *P*<.001). One difference that comes to mind could contribute to these findings. The profiles of the two physicians differed in two aspects, tendency and presence of reviews, and presence exerts an influence distinct from that of tendency, which might explain part of what appears as tendency effects in our analysis. Testing this must be left to further research. At least the stronger effect of the positive versus the negative review on physician choice corresponds well to the limitation of the dampening effect by health literacy to the group that read the negative review. If health literacy assuages the effect of negative reviews, it is no wonder that the effect of the positive review is stronger.

The major motive for this study was to see whether health literacy might dampen the effects of reviews written and uploaded by health care consumers, who might just not know enough to assess the medical side of a physician’s performance. The answer is, “Yes, but…” The dampening effect was found for subjective health literacy only. Our bet beforehand was that the expected effect was more likely to occur with performance-based health literacy measures.

The background for the distinction between performance-based and perception-based health literacy is the increasing scholarly insight that health literacy is a two-faced concept that contains not only willingness to participate in health decisions but also the capability to do it. Increasing patient participation in health care will be beneficial only if patients are not only willing but also capable of playing a more active role. If you give patients more of a say in their own health care while they lack the necessary capabilities, health decisions might worsen. The relationship between the two is often blurred, but it is imperative to distinguish them properly [75].

Perception-based measures of health literacy are associated with motivation and willingness, while performance-based measures aim at the objective side, at ability. Therefore we had, as said, the bet on the performance-based NVS measure as moderating the effects of patient-written physician reviews negatively. For the perception-based measures, an explanation close at hand might be that some transfer occurred. Higher values on these measures would indicate an overestimation not only of one’s own but also of other people’s communication competence. As a consequence, there would be a positive moderation effect of subjective health literacy rather than the negative effect we find.

Diverging results from applying these measures raise the question of whether the objective, performance-based indicators do indeed measure the same concept as the subjective, perception-based indicators do [76]. The lack of any correlation between the two measures supports the assumption of two concepts.

The finding of different effects of health literacy on preference for Dr Müller can be explained in various ways. More literate people, particularly those who consider themselves as being more familiar with health information on the internet, are on a general level more experienced with negative reviews (not just in the context of health) and less willing to take negative information at face value. Overall, negative reviews are quite common. A presentation of the doctor as “Quite nice, but he doesn’t really have a clue,” combined with the critique that the doctor did not properly examine the patient and prescribed the wrong medication impressed literate people only to a minor degree. This reasoning could be challenged by the fact that most reviews on PRWs are positive.

Another explanation is that people with higher literacy skills consider themselves more capable of constructively dealing with doctors who treat them in a deficient way. Showing a higher level of self-efficacy may make literate people believe they can handle critical situations as presented in the negative review. In one word, they are less afraid of the impact a negative review might have on them, while for less literate people, the negative effect looms larger, to use the description of the value curve from prospect theory [77]. This second point implies there is no moderation effect of the NVS, the other indicator of health literacy used in this study. That is to say, this performance-based measure, other than the perception-based eHEALS measure, is free of any self-efficacy component.

If health literacy, in spite of our results, should have a potential to increase skepticism of physician reviews written by health care consumers and published anonymously, then some of this potential should show in our interview data beyond those used in the study so far. Usefulness and skepticism toward PRWs correlate with objective and subjective health literacy differently. Perceived skepticism of eWOM in the field of health, for instance, is negatively correlated with the perception of the usefulness of PRWs (*r*=–.485, *P*<.001). That means people who are skeptical of PRWs consequently find them less useful. Performance-based health literacy is not at all correlated with skepticism (*r*=–.02, *P*=.80) or perceived usefulness (*r*=.04, *P*=.54). That means it is not true, as one could assume, that people with an objectively higher level of health literacy get more skeptic of PRWs and find them less useful. To the contrary, people found to score high on perception-based health literacy find PRWs more useful (*r*=.22, *P*<.001) and are less skeptical of them (*r*=–.21, *P*<.001), which stands in contrast to our finding that they are less willing to follow the latent advice contained in reviews. All in all, there is not much reason to assume health literacy can contribute to solve a problem it has helped create: a more autonomous patient who at times is not equipped with the abilities and knowledge necessary to get involved in decision making.

The subject of this research, PRWs and their role in physician choice, is different from most other health decisions that are studied. Health decisions are usually demanded by a patient who is ill or has some prevention concern. The doctor provides the good, but under the rule of participation policies, asks and/or encourages the patient to get involved. If the wrong decision is made, that will primarily be injurious to the patient. Physician choice is a decision the patient makes on their own, at least there is usually no physician involved. The primary damage is the physician’s—the loss of business—and the decision need not even be wrong. Wrong physician choice decisions can also damage the patient if there is a wide qualification differential between the physicians the patient does not notice or heed. One would think such cases are the exception rather than the rule. If physicians are concerned about choice decisions not being based on expertise, their concern might be fueled by the fact that they can be the victim of unreasonable patient decisions themselves. If patient input in usual health care decisions is suboptimal, the damage is the patient’s and the doctor is there to correct. It is no big surprise that physicians are less concerned about the quality of patient input in these cases.

Patients are skeptical of their ability to make judgments on the professional expertise of physicians and yet still pay attention to and draw conclusions from them. With PRWs, however, health care consumers talk to people who are just like themselves. That is a rare thing or was until the internet and especially Web 2.0 apps. “I might have some qualms about my and my fellow patients’ ability to rate doctors, but I share with them all the goodwill, all my experience with doctors, and a lot of suffering when doctors could not help.” Feelings like these might indirectly give physician ratings some legitimacy and value in the eyes of patients.

As to the results on the moderation effect of health literacies, they foremost suggest that performance-based and perception-based health literacies are to be separated more strictly than has been done in the past. One of the drawbacks of patient participation policy might well be that some patients actually lack a basis for adequate interference or involvement but still insist on having a say in medical decisions in the widest sense. It is this group that might be most at risk of suffering detrimental consequences.

### Limitations

This study faces limitations concerning its design and data collection. Specifically, the data were collected via an online platform where participants take part in studies to gain rewards (eg, vouchers, airline miles). Hence, it cannot be ruled out that such data collection attracts a specific audience. Even though our sample corresponded to the Swiss population in terms of education, age, and gender [59], results should not be generalized. Additionally, in order to check the attention and comprehension of the participants, we automatically screened out individuals who did not pass the manipulation check, in which they were asked about the number of text reviews present on each of the two physician profiles. By these means we ensured that the review on the assessment of the doctor’s competence was noticed. As the contracting firm Qualtrics removed those participants automatically, we are not able to report on the number of individuals who did not pass the manipulation check.

Another limitation of the study was based on randomization. Even though we randomized the order in which the two physician profiles were presented, we did not randomize the order of the text reviews on Dr Müller’s profile. This may have impacted participant choice due to primacy effects [62]. In order to account for primacy effects, future studies should control for this or randomize the order.

In addition, the subjective health literacy measure explicitly aims at online capabilities and is thus substantially close to online physician rating, while the objective measure of health literacy by understanding a food label has no such proximity. The subjective measure better applies to the context of online information and credibility judgments than does the NVS. So if people are not completely amiss in assessing their own skills, the relationship between high eHEALS scores and reasonable reaction to one bad review of Dr Müller might be the consequence of the closeness of the situation and measure rather than the subjective nature of the measure. In hindsight, it was probably not a good idea to employ only 7 of the 8 items on the eHEALS, although we doubt that the omission of one item in an 8-item measure would have changed the substance of the analysis much.

### Conclusion

Text reviews that assess a physician’s competence weigh heavily on the choice of physician on PRWs, even though health care consumers have voiced skepticism toward the truthfulness of such reviews and doubt their own and fellow patients’ capabilities to assess health care and physicians accurately [28,29]. Our study evidenced that patient-written reviews impact health care consumer choices and attitudes toward a physician and affect their perceptions of the doctor’s skill and trustworthiness. These findings sustain physician worries that even one negative PRW review can be highly damaging to their reputation [71]. Furthermore, these results put forward that internet users select health care providers based on patient-written reviews that contain information only weakly, selectively, or not at all related to objective measures of care quality according to various studies [24,78]. Hopes that health literacy might raise awareness of the insufficient basis of the rating of physicians’ medical performance by patients have mostly not been sustained. Rather, the divergence of subjective and objective health literacy might compound the problem.

## Appendix

Multimedia Appendix 1 Instructions presented to participants before viewing the physician profiles (translated from German).

Multimedia Appendix 2 Questionnaire examining users' response to physicians' reviews and rating on websites.

## Abbreviations

ANOVA: analysis of variance
eHEALS: eHealth Literacy Scale
eWOM: electronic word of mouth
NVS: newest vital sign
PRW: physician rating website
