# Supplementary material for: Influence of Health Literacy on Effects of Patient Rating Websites: Survey Study Using a Hypothetical Situation and Fictitious Doctors
Source: J Med Internet Res. 2020 Apr 6;22(4):e14134. doi: 10.2196/14134 (PMC7171560; doi:10.2196/14134)
Supplement: Multimedia Appendix 1 [file jmir_v22i4e14134_app1.docx]

Multimedia Appendix 1. Instructions presented to participants before viewing the physician profiles (translated from German).

*“Imagine that you moved to a new town called Freising (a small city approximately 40km North-East of Munich). During the move, when setting up your furniture, your foot was scratched by a nail.*

*A couple of days later, you notice that the wound got infected and that your foot is heavily swollen. You know that you now urgently need to visit a physician.*

*As you only recently moved to Freising and have not yet met anyone in town, you decide to go online to search for a doctor who could help you. You find out that there are two physicians relatively close to your home: Dr. Müller and Dr. Schmidt.*

*Please read carefully through their profiles, which are displayed on the next page. Subsequently, you will be asked some questions about them.”*
