# Supplementary material for: Influence of Health Literacy on Effects of Patient Rating Websites: Survey Study Using a Hypothetical Situation and Fictitious Doctors
Source: J Med Internet Res. 2020 Apr 6;22(4):e14134. doi: 10.2196/14134 (PMC7171560; doi:10.2196/14134)
Supplement: Multimedia Appendix 2 [file jmir_v22i4e14134_app2.docx]

Multimedia Appendix 2. Questionnaire: Final_Experiment Physician Rating Websites 07.03.2017

Q1.1 Liebe Teilnehmerin, lieber Teilnehmer

Diese Studie erforscht Arztbewertungswebseiten im Internet. Auf solchen Webseiten können Nutzer einen Arzt suchen und Bewertungen lesen, welche von Patienten abgegeben wurden. Diese Studie untersucht, wie diese Webseiten genutzt werden.

Informationen zur Studie

Die Teilnahme an dieser Studie dauert ca. 20 Minuten und besteht aus drei Teilen:

1. Zuerst werden Sie gebeten, einige Fragen zu Ihrer Nutzung von Bewertungswebseiten zu beantworten.

2. Anschliessend werden Sie zwei Arztprofile durchlesen.

3. Danach werden Sie zu Ihrem Eindruck der gesehenen Informationen befragt.

Bitte beantworten Sie alle Fragen so ehrlich wie möglich.

Teilnahmebedingungen und Anonymität der Daten

Um an dieser Studie teilzunehmen, müssen Sie mindestens 18 Jahre alt und in der Schweiz wohnhaft sein. Alle in dieser Studie gesammelten Daten werden anonymisiert, ausschliesslich für wissenschaftliche Zwecke verwendet und nicht an Drittparteien weitergegeben. Sie können die Teilnahme an dieser Studie jederzeit und ohne Begründung abbrechen.

Diese Studie wurde von der Ethikkommission der Università della Svizzera italiana geprüft.

Wenn Sie diesen Fragebogen beginnen, erklären Sie sich mit den Teilnahmebedingungen einverstanden.

Für Ihre Teilnahme an dieser Studie bedanken wir uns schon im Voraus.

Bei Fragen wenden Sie sich bitte an:

Fabia Rothenfluh, M.Sc. (Doktorandin)

Prof. Dr. Peter J. Schulz (Projektleiter)

Institut für Kommunikation und Gesundheit (ICH)

Università della Svizzera italiana, Lugano (USI)

CH-6904 Lugano

Tel: (+41) 058 666 4485

Q2.1 Viele kommerzielle Webseiten bieten Benotungen und Bewertungen für Produkte an. So können sich Kunden vor dem Kauf über das Produkt informieren. Webseiten und Firmen, die solche Bewertungen anbieten sind beispielsweise booking.com, trivago.com, amazon.com, ricardo.ch, ebay.com, zalando.ch.

Bitte beantworten Sie nun folgende Fragen zu Ihrer Nutzung solcher Webseiten.

Q2.2 Wie oft lesen Sie vor dem Kauf eines neuen Produkts Bewertungen oder Benotungen dazu im Internet nach?

- Nie (1)
- Selten (2)
- Manchmal (3)
- Oft (4)
- Immer (5)

Q2.3 Wie häufig haben Sie bereits selbst eine Bewertung verfasst?

- Nie (1)
- 1-3 mal (2)
- 4-10 mal (3)
- Mehr als 10 mal (4)

Q2.4 Nun möchten wir Ihnen einige Fragen zu Ihrer Suche nach Ärzten im Internet stellen. Wie häufig haben Sie im Internet nach einem Arzt oder einer Ärztin gesucht?

- Nie (1)
- 1-3 mal (2)
- 4-10 mal (3)
- Mehr als 10 mal (4)

Q2.5 Wie oft haben Sie Arztbewertungswebseiten zu Rate gezogen, um sich über einen Arzt oder eine Ärztin zu informieren?

- Nie (1)
- 1-3 mal (2)
- 4-10 mal (3)
- Mehr als 10 mal (4)

Q2.6 Wie oft haben Sie selbst eine Arztbewertung verfasst?

- Nie (1)
- 1-3 mal (2)
- 4-10 mal (3)
- Mehr als 10 mal (4)

Q2.7 Welche der folgenden Bewertungswebseiten kennen Sie? (Mehrere Antworten möglich)

- Ich kenne keine Arztbewertungswebseiten (1)
- www.okdoc.ch (2)
- www.zahnarztvergleich.ch (3)
- www.doktor.ch (4)
- www.docapp.ch (5)
- www.medicosearch.ch (6)
- www.jameda.de (7)
- www.weisseseiten.de (8)
- Andere (bitte einfügen): (9) ____________________

Display This Question:

If Welche der folgenden Bewertungswebseiten kennen Sie? (bitte klicken Sie alle an, die Sie kennen) www.okdoc.ch Is Selected

Or Welche der folgenden Bewertungswebseiten kennen Sie? (bitte klicken Sie alle an, die Sie kennen) www.zahnarztvergleich.ch Is Selected

Or Welche der folgenden Bewertungswebseiten kennen Sie? (bitte klicken Sie alle an, die Sie kennen) www.doktor.ch Is Selected

Or Welche der folgenden Bewertungswebseiten kennen Sie? (bitte klicken Sie alle an, die Sie kennen) www.docapp.ch Is Selected

Or Welche der folgenden Bewertungswebseiten kennen Sie? (bitte klicken Sie alle an, die Sie kennen) www.medicosearch.ch Is Selected

Or Welche der folgenden Bewertungswebseiten kennen Sie? (bitte klicken Sie alle an, die Sie kennen) www.jameda.de Is Selected

Or Welche der folgenden Bewertungswebseiten kennen Sie? (bitte klicken Sie alle an, die Sie kennen) www.weisseseiten.de Is Selected

Or Welche der folgenden Bewertungswebseiten kennen Sie? (bitte klicken Sie alle an, die Sie kennen) Andere (bitte einfügen): Is Selected

Q2.8 Wie häufig nutzen Sie die oben angekreuzte(n) Arztbewertungswebseite(n)?

- Fast nie (1)
- Selten (2)
- Manchmal (3)
- Oft (4)
- Sehr oft (5)

Q3.1 Stellen Sie sich vor, Sie wären nach Berlin umgezogen. Während Ihres Umzugs, als Sie die Möbel aufbauten, zogen Sie sich am Fuss einen Kratzer durch einen Nagel zu.

Ein paar Tage später stellen Sie fest, dass sich die Wunde infiziert hat und ihr Fuss stark angeschwollen ist. Sie wissen, dass Sie nun umgehend einen Arzt aufsuchen müssen.

Weil Sie erst kurz in der Stadt wohnen und noch niemanden kennen, entscheiden Sie sich im Internet nach einem Arzt zu suchen, der Ihnen helfen könnte. Sie finden heraus, dass es in Ihrer Nähe zwei Ärzte gibt, die Sie aufsuchen könnten: Dr. Müller und Dr. Schmidt.

Bitte lesen Sie sich auf der nächsten Seite die Profile beider Ärzte gut durch. Anschliessend werden Ihnen einige Fragen dazu gestellt.

Q4.1


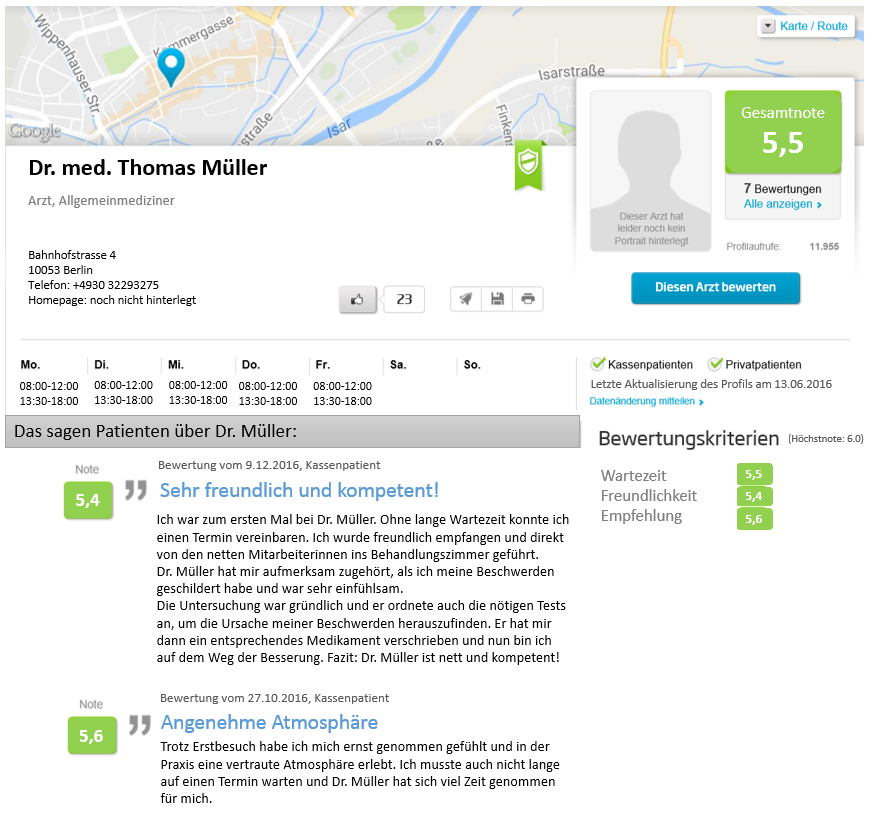


Q4.2


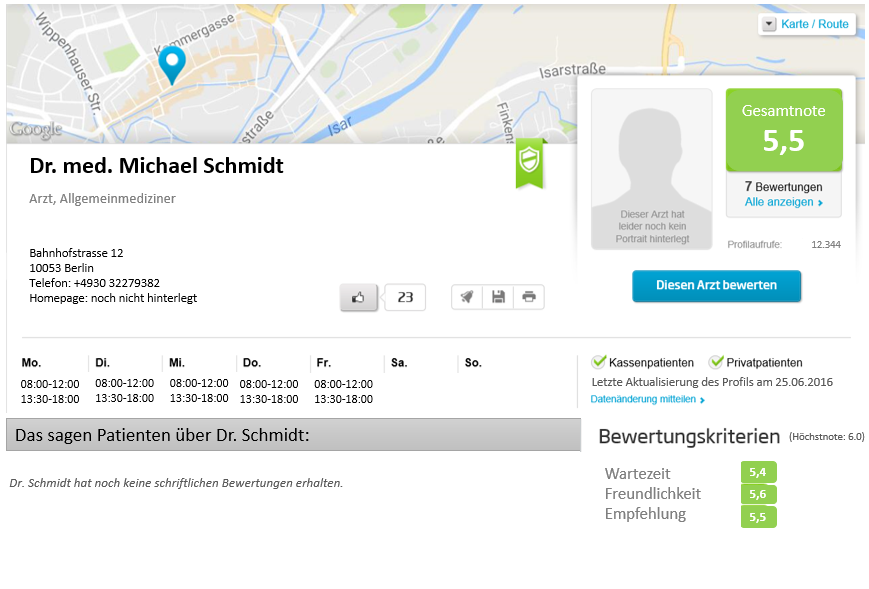


Q4.3 Nachdem Sie die beiden Profile der Ärzte durchgelesen haben, welchen Arzt würden Sie konsultieren?

- Definitiv Dr. Müller (1)
- Wahrscheinlich Dr. Müller (2)
- Eher Dr. Müller (3)
- Weder Dr. Müller noch Dr. Schmidt (4)
- Eher Dr. Schmidt (5)
- Wahrscheinlich Dr. Schmidt (6)
- Definitiv Dr. Schmidt (7)

Q4.4


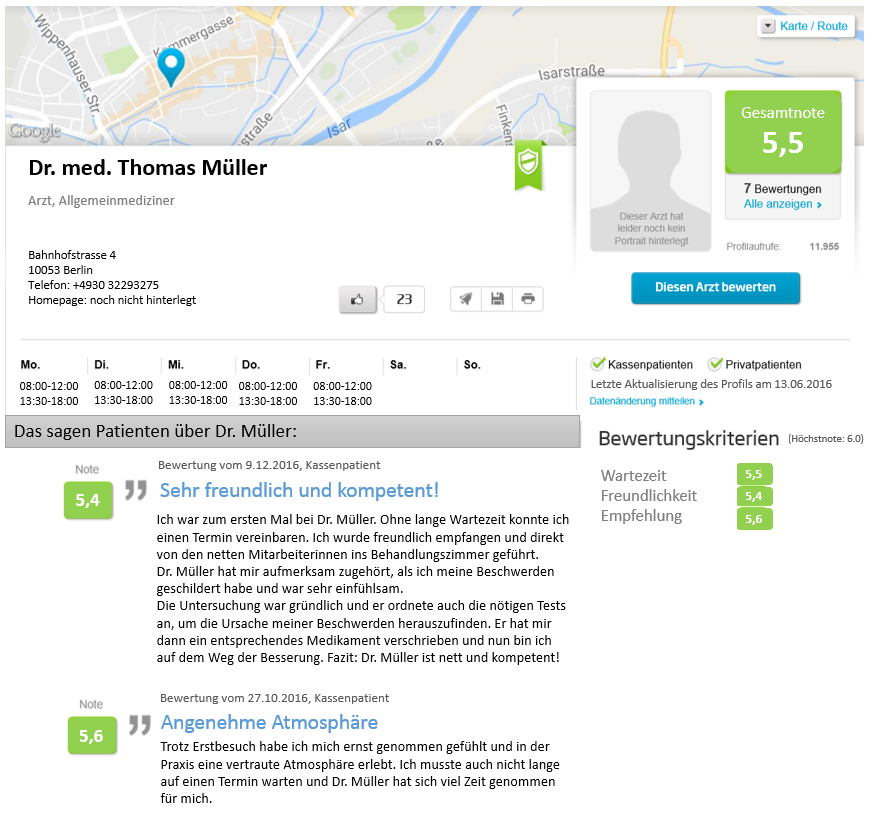


Q4.5 Bitte beurteilen Sie das Profil von Dr. Müller:

|  | 1 (1) | 2 (2) | 3 (3) | 4 (4) | 5 (5) |
| --- | --- | --- | --- | --- | --- |
| Hilfreich:Nutzlos (Q14.1_1) |  |  |  |  |  |
| Informativ:Verwirrend (Q14.1_2) |  |  |  |  |  |
| Glaubwürdig:Unglaubwürdig (Q14.1_3) |  |  |  |  |  |
| Vertrauenserweckend:Irreführend (Q14.1_4) |  |  |  |  |  |
| Verlässlich:Unzuverlässig (Q14.1_5) |  |  |  |  |  |
| Langweilig:Interessant (Q14.1_6) |  |  |  |  |  |
| Übersichtlich:Ungeordnet (Q14.1_7) |  |  |  |  |  |
| Realistisch:Verfälscht (Q14.1_8) |  |  |  |  |  |

Q4.6 Bitte geben Sie an, wie Sie Dr. Müller nach dem Lesen der online-Bewertung, beurteilen:

|  | 1 (1) | 2 (2) | 3 (3) | 4 (4) | 5 (5) | 6 (6) | 7 (7) |
| --- | --- | --- | --- | --- | --- | --- | --- |
| Dr. Müller macht einen guten Eindruck auf mich. (1) |  |  |  |  |  |  |  |
| Dr. Müller überzeugt mich. (2) |  |  |  |  |  |  |  |
| Ich würde Dr. Müller vertrauen. (3) |  |  |  |  |  |  |  |

Q4.7


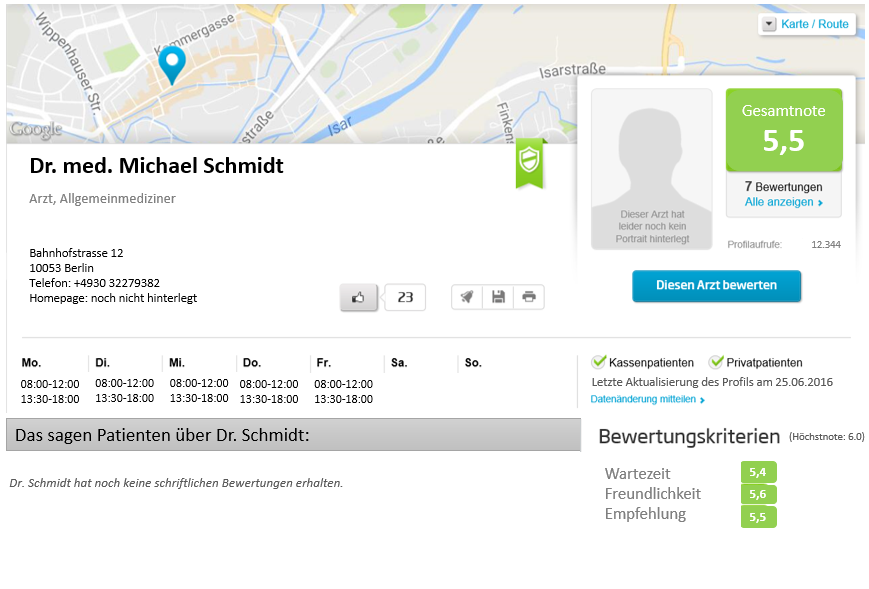


Q4.8 Bitte beurteilen Sie das Profil von Dr. Schmidt:

|  | 1 (1) | 2 (2) | 3 (3) | 4 (4) | 5 (5) |
| --- | --- | --- | --- | --- | --- |
| Hilfreich:Nutzlos (Q14.1_1) |  |  |  |  |  |
| Informativ:Verwirrend (Q14.1_2) |  |  |  |  |  |
| Glaubwürdig:Unglaubwürdig (Q14.1_3) |  |  |  |  |  |
| Vertrauenserweckend:Irreführend (Q14.1_4) |  |  |  |  |  |
| Verlässlich:Unzuverlässig (Q14.1_5) |  |  |  |  |  |
| Langweilig:Interessant (Q14.1_6) |  |  |  |  |  |
| Übersichtlich:Ungeordnet (Q14.1_7) |  |  |  |  |  |
| Realistisch:Verfälscht (Q14.1_8) |  |  |  |  |  |

Q4.9 Bitte geben Sie an, wie Sie Dr. Schmidt nach dem Lesen der online-Bewertung beurteilen:

|  | 1 (1) | 2 (2) | 3 (3) | 4 (4) | 5 (5) | 6 (6) | 7 (7) |
| --- | --- | --- | --- | --- | --- | --- | --- |
| Dr. Schmidt macht einen guten Eindruck auf mich. (1) |  |  |  |  |  |  |  |
| Dr. Schmidt überzeugt mich. (2) |  |  |  |  |  |  |  |
| Ich würde Dr. Schmidt vertrauen. (3) |  |  |  |  |  |  |  |

Q5.1


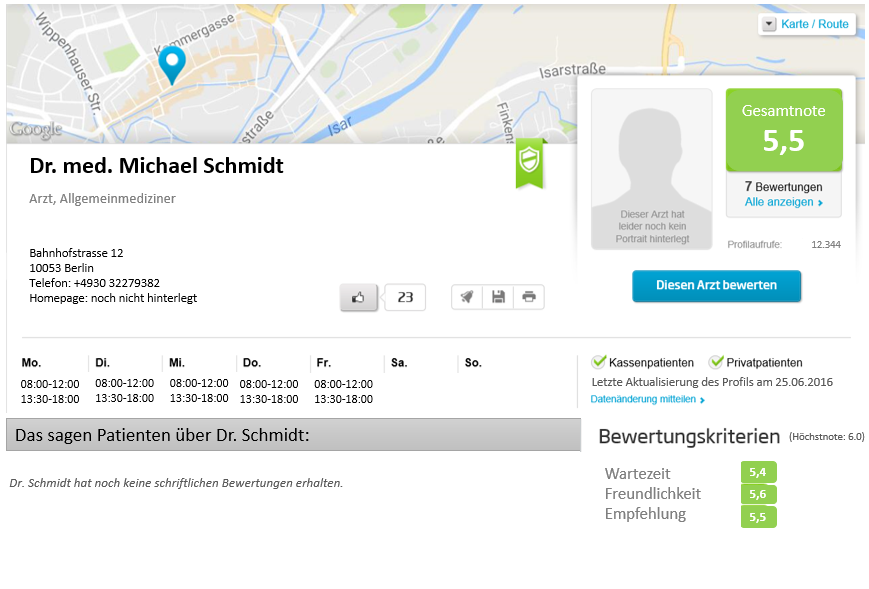


Q5.2


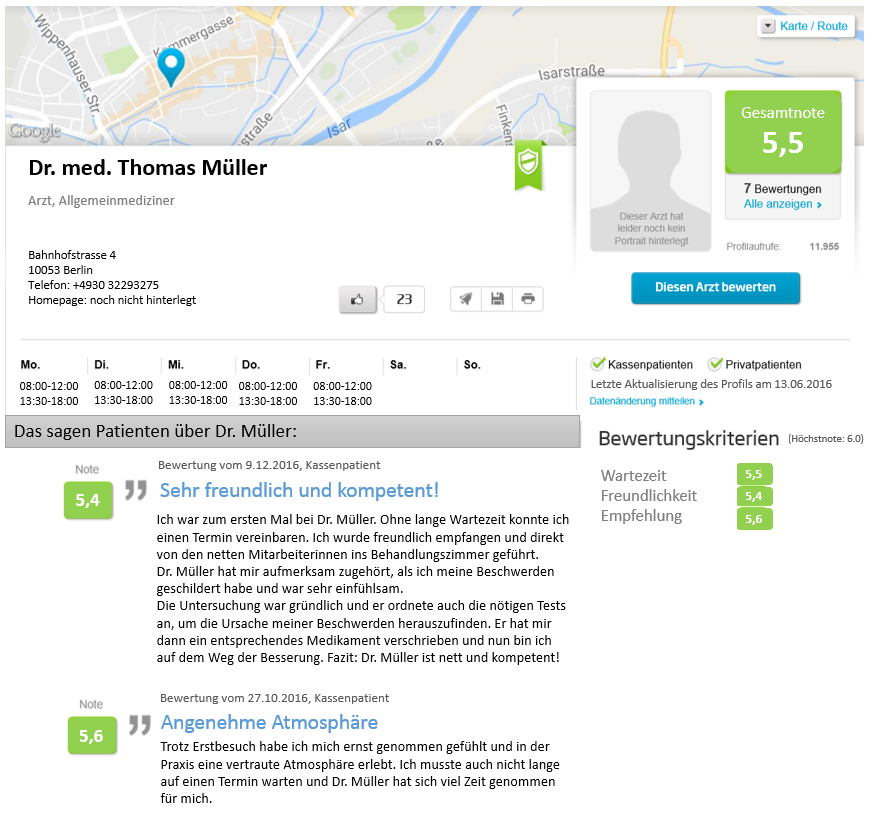


Q5.3 Nachdem Sie die beiden Profile der Ärzte durchgelesen haben, welchen Arzt würden Sie konsultieren?

- Definitiv Dr. Müller (1)
- Wahrscheinlich Dr. Müller (2)
- Eher Dr. Müller (3)
- Weder Dr. Müller noch Dr. Schmidt (4)
- Eher Dr. Schmidt (5)
- Wahrscheinlich Dr. Schmidt (6)
- Definitiv Dr. Schmidt (7)

Q5.4


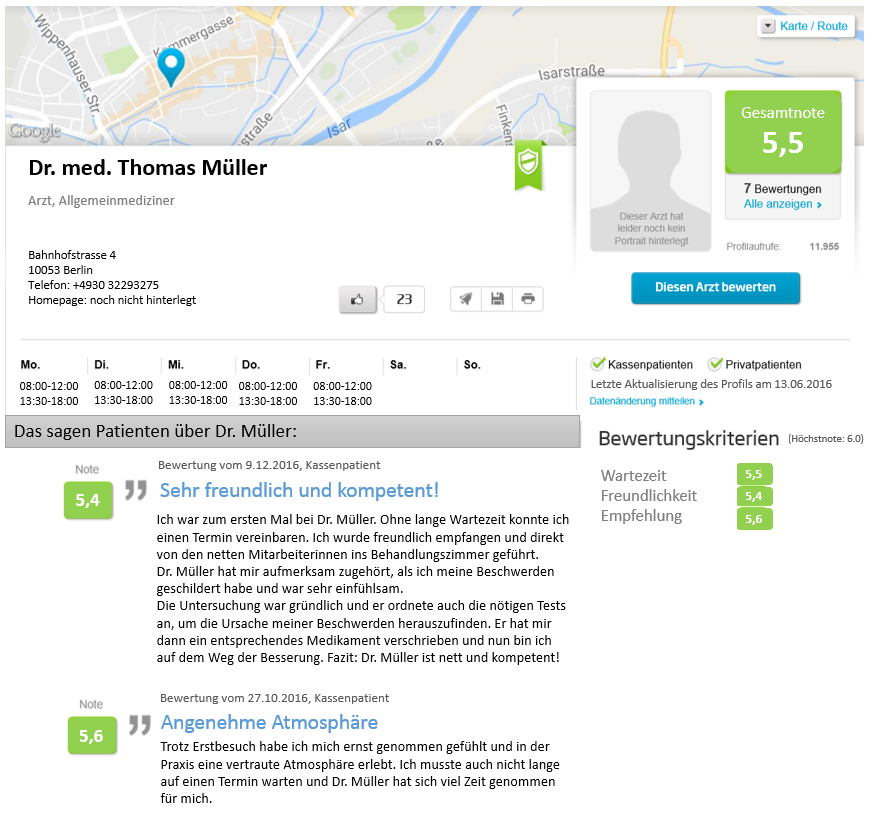


Q5.5 Bitte beurteilen Sie das Profil von Dr. Müller:

|  | 1 (1) | 2 (2) | 3 (3) | 4 (4) | 5 (5) |
| --- | --- | --- | --- | --- | --- |
| Hilfreich:Nutzlos (Q14.1_1) |  |  |  |  |  |
| Informativ:Verwirrend (Q14.1_2) |  |  |  |  |  |
| Glaubwürdig:Unglaubwürdig (Q14.1_3) |  |  |  |  |  |
| Vertrauenserweckend:Irreführend (Q14.1_4) |  |  |  |  |  |
| Verlässlich:Unzuverlässig (Q14.1_5) |  |  |  |  |  |
| Langweilig:Interessant (Q14.1_6) |  |  |  |  |  |
| Übersichtlich:Ungeordnet (Q14.1_7) |  |  |  |  |  |
| Realistisch:Verfälscht (Q14.1_8) |  |  |  |  |  |

Q5.6 Bitte geben Sie an, wie Sie Dr. Müller nach dem Lesen der online-Bewertung, beurteilen:

|  | 1 (1) | 2 (2) | 3 (3) | 4 (4) | 5 (5) | 6 (6) | 7 (7) |
| --- | --- | --- | --- | --- | --- | --- | --- |
| Dr. Müller macht einen guten Eindruck auf mich. (1) |  |  |  |  |  |  |  |
| Dr. Müller überzeugt mich. (2) |  |  |  |  |  |  |  |
| Ich würde Dr. Müller vertrauen. (3) |  |  |  |  |  |  |  |

Q5.7


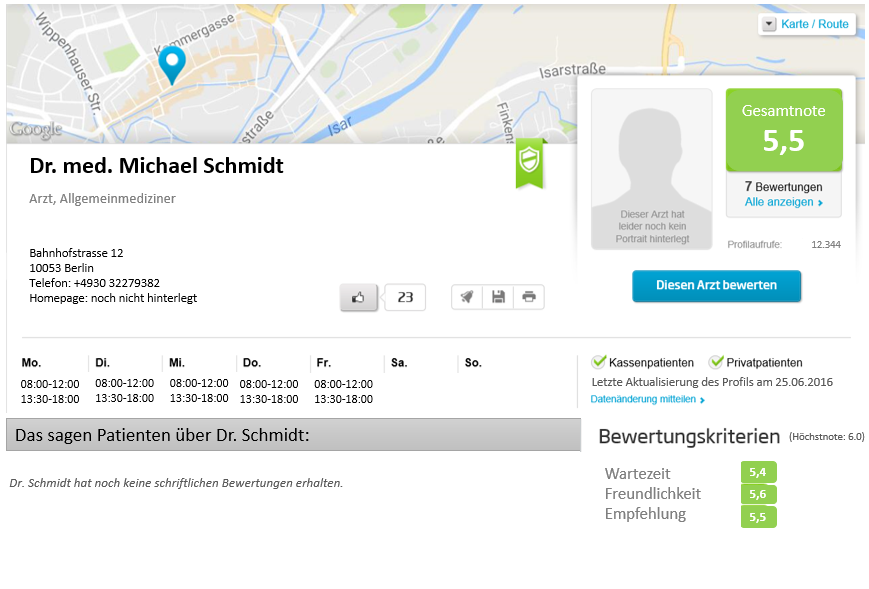


Q5.9 Bitte beurteilen Sie das Profil von Dr. Schmidt:

|  | 1 (1) | 2 (2) | 3 (3) | 4 (4) | 5 (5) |
| --- | --- | --- | --- | --- | --- |
| Hilfreich:Nutzlos (Q14.1_1) |  |  |  |  |  |
| Informativ:Verwirrend (Q14.1_2) |  |  |  |  |  |
| Glaubwürdig:Unglaubwürdig (Q14.1_3) |  |  |  |  |  |
| Vertrauenserweckend:Irreführend (Q14.1_4) |  |  |  |  |  |
| Verlässlich:Unzuverlässig (Q14.1_5) |  |  |  |  |  |
| Langweilig:Interessant (Q14.1_6) |  |  |  |  |  |
| Übersichtlich:Ungeordnet (Q14.1_7) |  |  |  |  |  |
| Realistisch:Verfälscht (Q14.1_8) |  |  |  |  |  |

Q5.8 Bitte geben Sie an, wie Sie Dr. Schmidt nach dem Lesen der online-Bewertung beurteilen:

|  | 1 (1) | 2 (2) | 3 (3) | 4 (4) | 5 (5) | 6 (6) | 7 (7) |
| --- | --- | --- | --- | --- | --- | --- | --- |
| Dr. Schmidt macht einen guten Eindruck auf mich. (1) |  |  |  |  |  |  |  |
| Dr. Schmidt überzeugt mich. (2) |  |  |  |  |  |  |  |
| Ich würde Dr. Schmidt vertrauen. (3) |  |  |  |  |  |  |  |

Q6.1


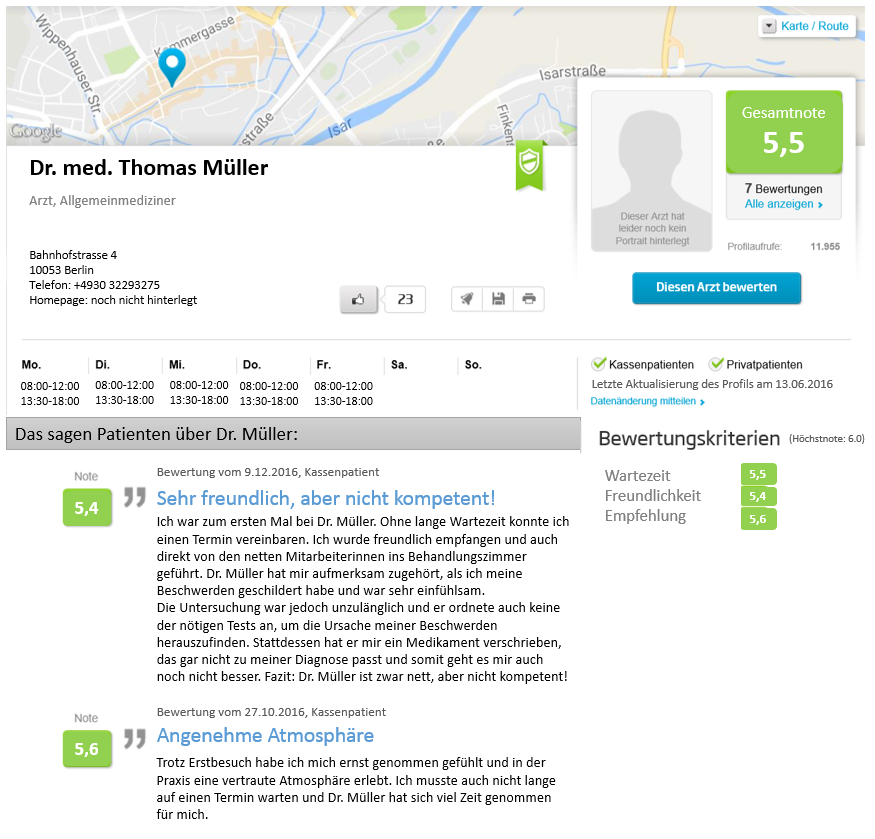


Q6.2


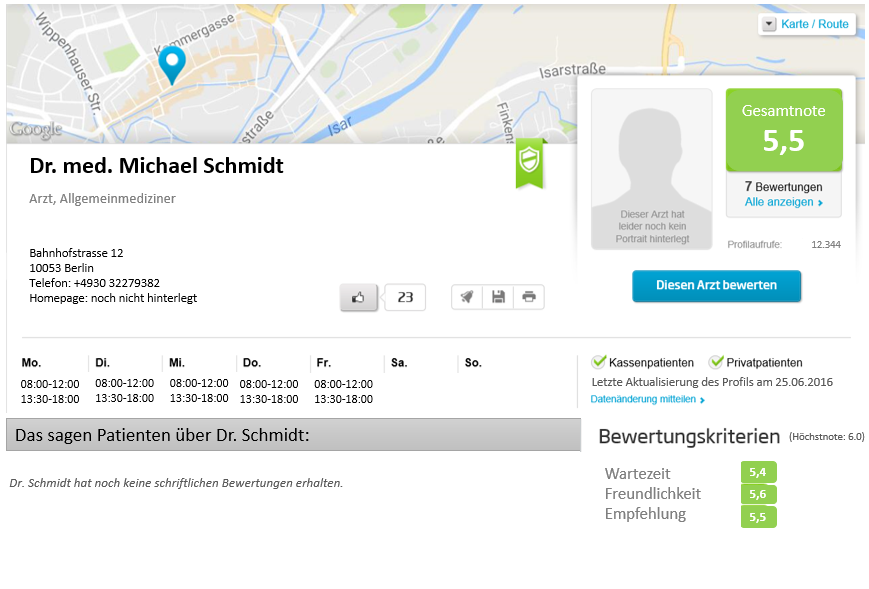


Q6.3 Nachdem Sie die beiden Profile der Ärzte durchgelesen haben, welchen Arzt würden Sie konsultieren?

- Definitiv Dr. Müller (1)
- Wahrscheinlich Dr. Müller (2)
- Eher Dr. Müller (3)
- Weder Dr. Müller noch Dr. Schmidt (4)
- Eher Dr. Schmidt (5)
- Wahrscheinlich Dr. Schmidt (6)
- Definitiv Dr. Schmidt (7)

Q6.4


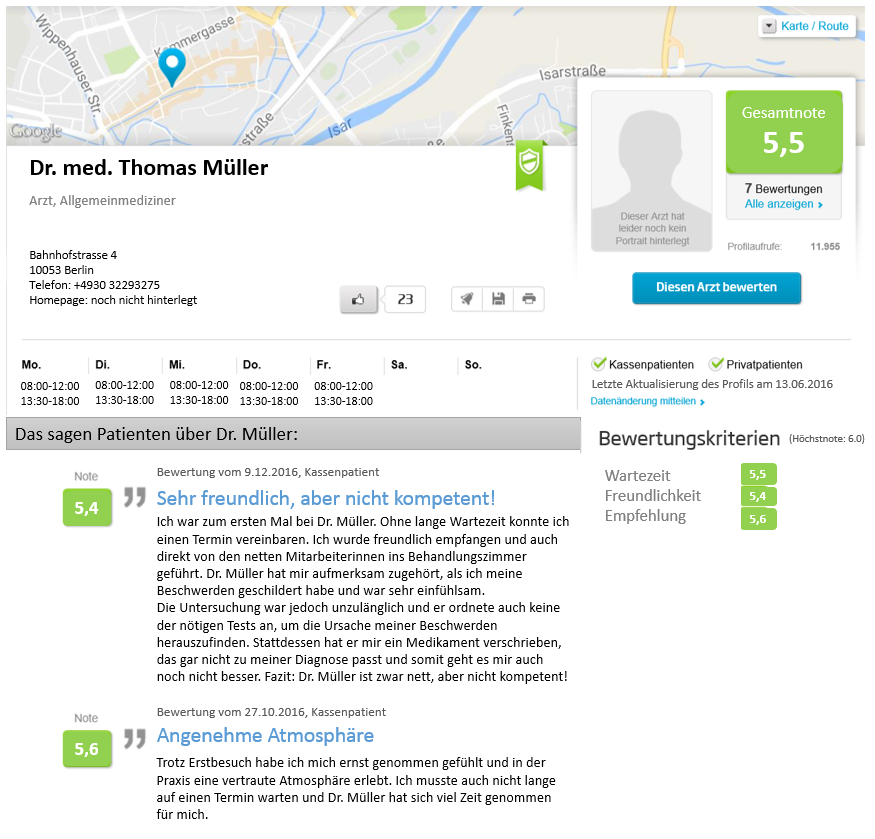


Q6.5 Bitte beurteilen Sie das Profil von Dr. Müller:

|  | 1 (1) | 2 (2) | 3 (3) | 4 (4) | 5 (5) |
| --- | --- | --- | --- | --- | --- |
| Hilfreich:Nutzlos (Q14.1_1) |  |  |  |  |  |
| Informativ:Verwirrend (Q14.1_2) |  |  |  |  |  |
| Glaubwürdig:Unglaubwürdig (Q14.1_3) |  |  |  |  |  |
| Vertrauenserweckend:Irreführend (Q14.1_4) |  |  |  |  |  |
| Verlässlich:Unzuverlässig (Q14.1_5) |  |  |  |  |  |
| Langweilig:Interessant (Q14.1_6) |  |  |  |  |  |
| Übersichtlich:Ungeordnet (Q14.1_7) |  |  |  |  |  |
| Realistisch:Verfälscht (Q14.1_8) |  |  |  |  |  |

Q6.6 Bitte geben Sie an, wie Sie Dr. Müller nach dem Lesen der online-Bewertung, beurteilen:

|  | 1 (1) | 2 (2) | 3 (3) | 4 (4) | 5 (5) | 6 (6) | 7 (7) |
| --- | --- | --- | --- | --- | --- | --- | --- |
| Dr. Müller macht einen guten Eindruck auf mich. (1) |  |  |  |  |  |  |  |
| Dr. Müller überzeugt mich. (2) |  |  |  |  |  |  |  |
| Ich würde Dr. Müller vertrauen. (3) |  |  |  |  |  |  |  |

Q6.7


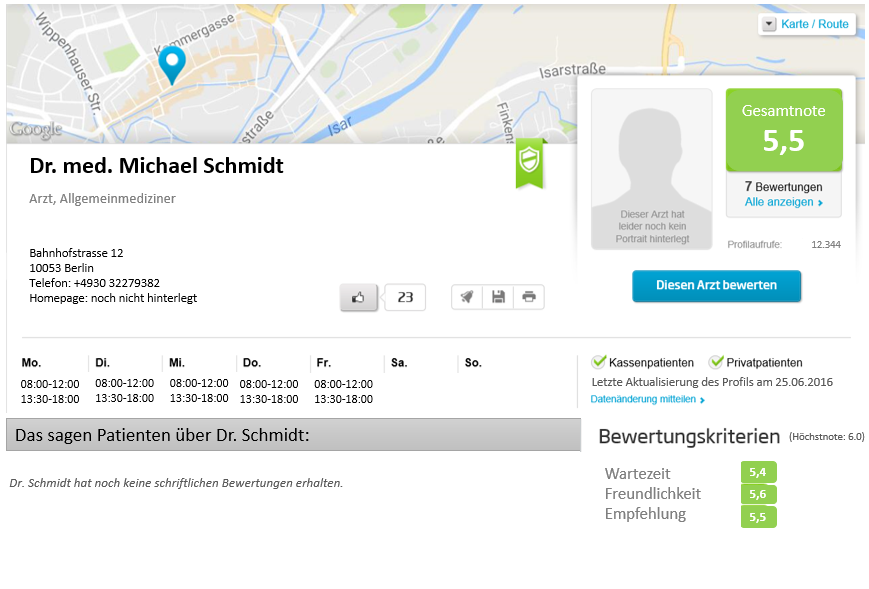


Q6.9 Bitte beurteilen Sie das Profil von Dr. Schmidt:

|  | 1 (1) | 2 (2) | 3 (3) | 4 (4) | 5 (5) |
| --- | --- | --- | --- | --- | --- |
| Hilfreich:Nutzlos (Q14.1_1) |  |  |  |  |  |
| Informativ:Verwirrend (Q14.1_2) |  |  |  |  |  |
| Glaubwürdig:Unglaubwürdig (Q14.1_3) |  |  |  |  |  |
| Vertrauenserweckend:Irreführend (Q14.1_4) |  |  |  |  |  |
| Verlässlich:Unzuverlässig (Q14.1_5) |  |  |  |  |  |
| Langweilig:Interessant (Q14.1_6) |  |  |  |  |  |
| Übersichtlich:Ungeordnet (Q14.1_7) |  |  |  |  |  |
| Realistisch:Verfälscht (Q14.1_8) |  |  |  |  |  |

Q6.8 Bitte geben Sie an, wie Sie Dr. Schmidt nach dem Lesen der online-Bewertung beurteilen:

|  | 1 (1) | 2 (2) | 3 (3) | 4 (4) | 5 (5) | 6 (6) | 7 (7) |
| --- | --- | --- | --- | --- | --- | --- | --- |
| Dr. Schmidt macht einen guten Eindruck auf mich. (1) |  |  |  |  |  |  |  |
| Dr. Schmidt überzeugt mich. (2) |  |  |  |  |  |  |  |
| Ich würde Dr. Schmidt vertrauen. (3) |  |  |  |  |  |  |  |

Q7.1


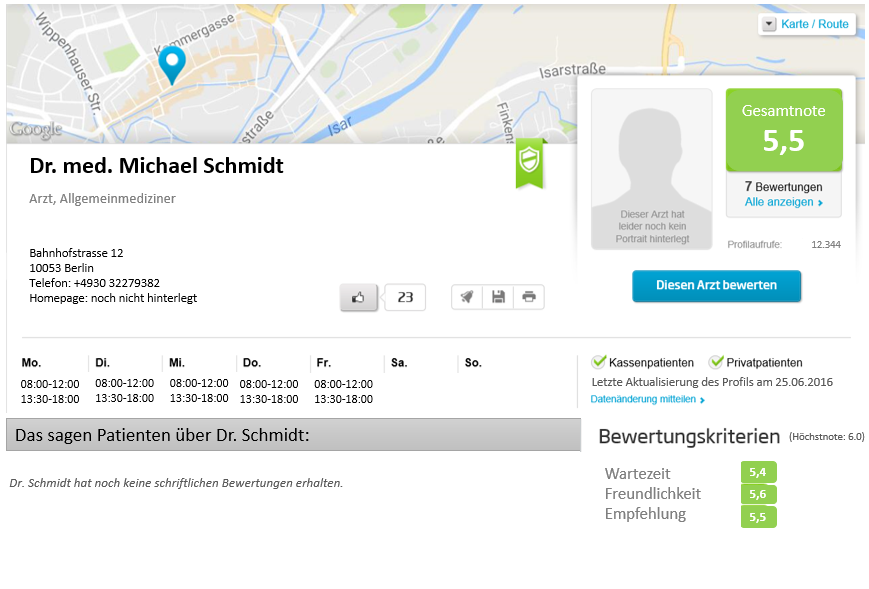


Q7.2


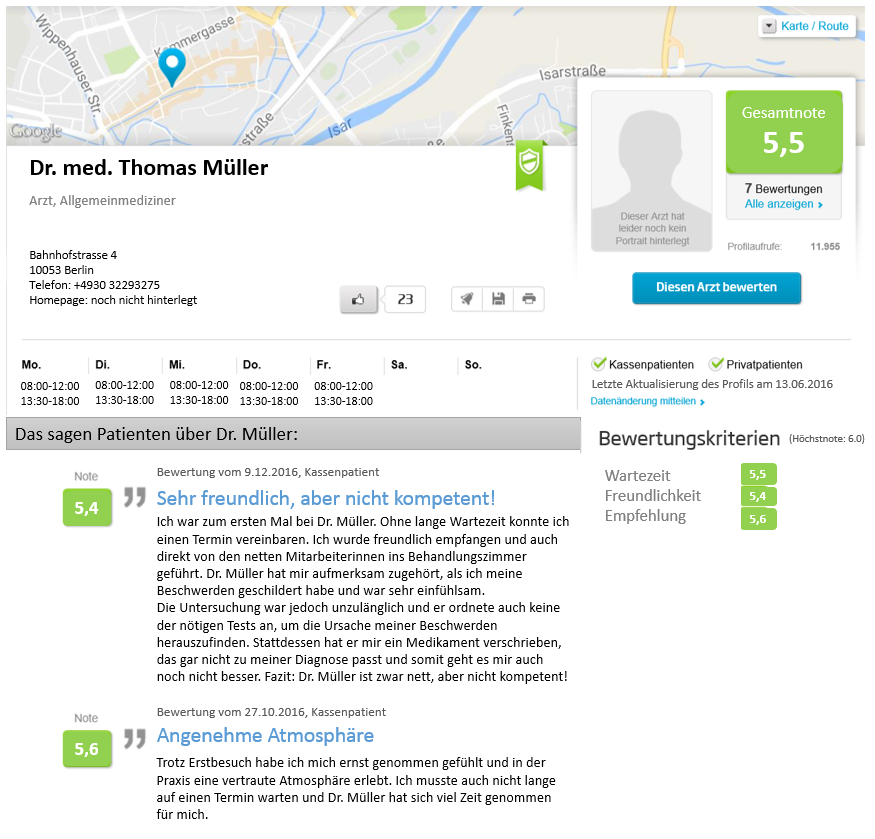


Q7.3 Nachdem Sie die beiden Profile der Ärzte durchgelesen haben, welchen Arzt würden Sie konsultieren?

- Definitiv Dr. Müller (1)
- Wahrscheinlich Dr. Müller (2)
- Eher Dr. Müller (3)
- Weder Dr. Müller noch Dr. Schmidt (4)
- Eher Dr. Schmidt (5)
- Wahrscheinlich Dr. Schmidt (6)
- Definitiv Dr. Schmidt (7)

Q7.4


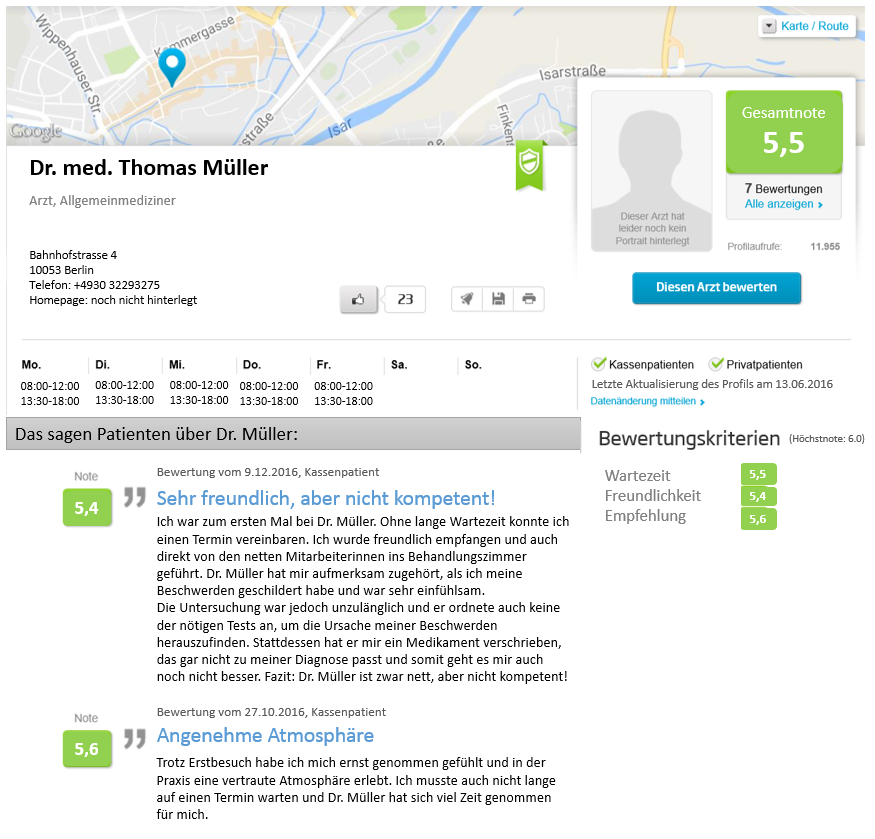


Q7.5 Bitte beurteilen Sie das Profil von Dr. Müller:

|  | 1 (1) | 2 (2) | 3 (3) | 4 (4) | 5 (5) |
| --- | --- | --- | --- | --- | --- |
| Hilfreich:Nutzlos (Q14.1_1) |  |  |  |  |  |
| Informativ:Verwirrend (Q14.1_2) |  |  |  |  |  |
| Glaubwürdig:Unglaubwürdig (Q14.1_3) |  |  |  |  |  |
| Vertrauenserweckend:Irreführend (Q14.1_4) |  |  |  |  |  |
| Verlässlich:Unzuverlässig (Q14.1_5) |  |  |  |  |  |
| Langweilig:Interessant (Q14.1_6) |  |  |  |  |  |
| Übersichtlich:Ungeordnet (Q14.1_7) |  |  |  |  |  |
| Realistisch:Verfälscht (Q14.1_8) |  |  |  |  |  |

Q7.6 Bitte geben Sie an, wie Sie Dr. Müller nach dem Lesen der online-Bewertung, beurteilen:

|  | 1 (1) | 2 (2) | 3 (3) | 4 (4) | 5 (5) | 6 (6) | 7 (7) |
| --- | --- | --- | --- | --- | --- | --- | --- |
| Dr. Müller macht einen guten Eindruck auf mich. (1) |  |  |  |  |  |  |  |
| Dr. Müller überzeugt mich. (2) |  |  |  |  |  |  |  |
| Ich würde Dr. Müller vertrauen. (3) |  |  |  |  |  |  |  |

Q7.7


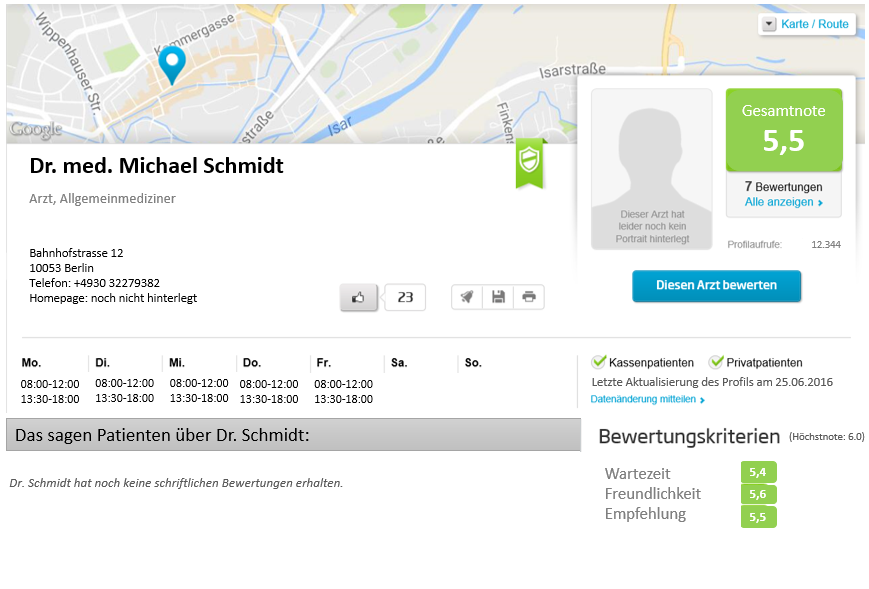


Q7.9 Bitte beurteilen Sie das Profil von Dr. Schmidt:

|  | 1 (1) | 2 (2) | 3 (3) | 4 (4) | 5 (5) |
| --- | --- | --- | --- | --- | --- |
| Hilfreich:Nutzlos (Q14.1_1) |  |  |  |  |  |
| Informativ:Verwirrend (Q14.1_2) |  |  |  |  |  |
| Glaubwürdig:Unglaubwürdig (Q14.1_3) |  |  |  |  |  |
| Vertrauenserweckend:Irreführend (Q14.1_4) |  |  |  |  |  |
| Verlässlich:Unzuverlässig (Q14.1_5) |  |  |  |  |  |
| Langweilig:Interessant (Q14.1_6) |  |  |  |  |  |
| Übersichtlich:Ungeordnet (Q14.1_7) |  |  |  |  |  |
| Realistisch:Verfälscht (Q14.1_8) |  |  |  |  |  |

Q7.8 Bitte geben Sie an, wie Sie Dr. Schmidt nach dem Lesen der online-Bewertung beurteilen:

|  | 1 (1) | 2 (2) | 3 (3) | 4 (4) | 5 (5) | 6 (6) | 7 (7) |
| --- | --- | --- | --- | --- | --- | --- | --- |
| Dr. Schmidt macht einen guten Eindruck auf mich. (1) |  |  |  |  |  |  |  |
| Dr. Schmidt überzeugt mich. (2) |  |  |  |  |  |  |  |
| Ich würde Dr. Schmidt vertrauen. (3) |  |  |  |  |  |  |  |

Q8.1


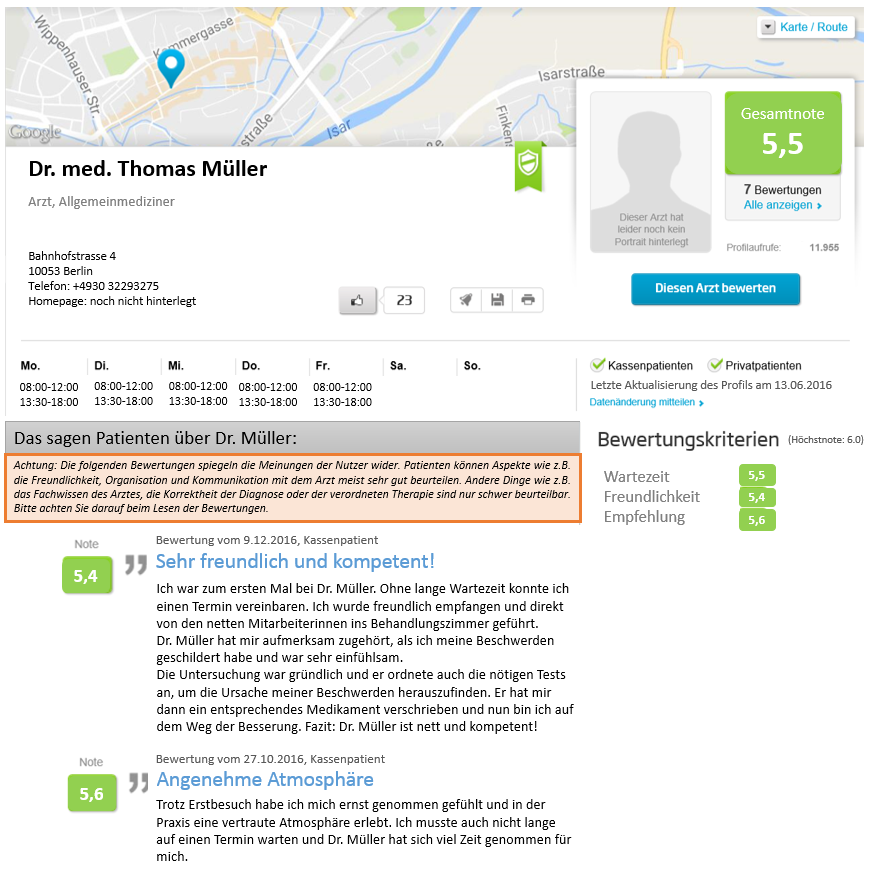


Q8.2


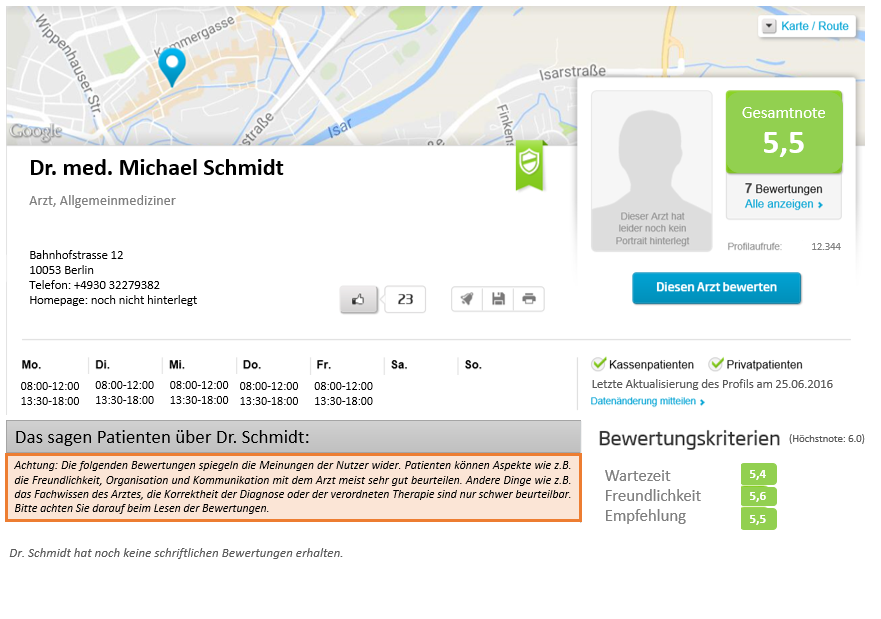


Q8.3 Nachdem Sie die beiden Profile der Ärzte durchgelesen haben, welchen Arzt würden Sie konsultieren?

- Definitiv Dr. Müller (1)
- Wahrscheinlich Dr. Müller (2)
- Eher Dr. Müller (3)
- Weder Dr. Müller noch Dr. Schmidt (4)
- Eher Dr. Schmidt (5)
- Wahrscheinlich Dr. Schmidt (6)
- Definitiv Dr. Schmidt (7)

Q8.4


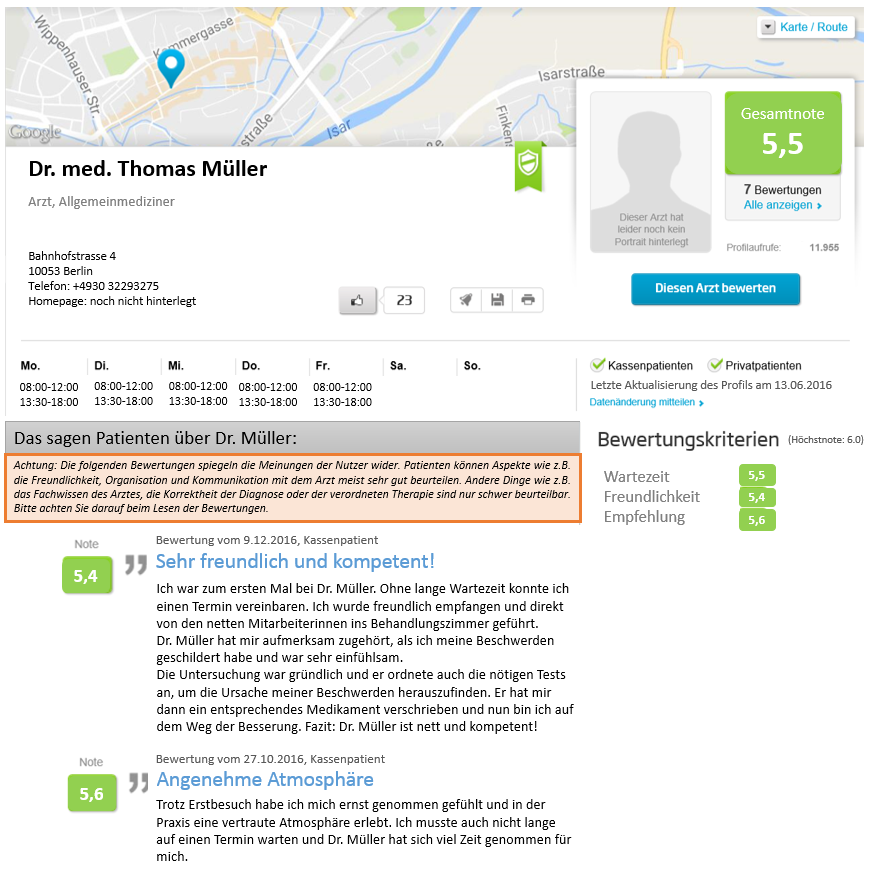


Q8.5 Bitte beurteilen Sie das Profil von Dr. Müller:

|  | 1 (1) | 2 (2) | 3 (3) | 4 (4) | 5 (5) |
| --- | --- | --- | --- | --- | --- |
| Hilfreich:Nutzlos (Q14.1_1) |  |  |  |  |  |
| Informativ:Verwirrend (Q14.1_2) |  |  |  |  |  |
| Glaubwürdig:Unglaubwürdig (Q14.1_3) |  |  |  |  |  |
| Vertrauenserweckend:Irreführend (Q14.1_4) |  |  |  |  |  |
| Verlässlich:Unzuverlässig (Q14.1_5) |  |  |  |  |  |
| Langweilig:Interessant (Q14.1_6) |  |  |  |  |  |
| Übersichtlich:Ungeordnet (Q14.1_7) |  |  |  |  |  |
| Realistisch:Verfälscht (Q14.1_8) |  |  |  |  |  |

Q8.6 Bitte geben Sie an, wie Sie Dr. Müller nach dem Lesen der online-Bewertung, beurteilen:

|  | 1 (1) | 2 (2) | 3 (3) | 4 (4) | 5 (5) | 6 (6) | 7 (7) |
| --- | --- | --- | --- | --- | --- | --- | --- |
| Dr. Müller macht einen guten Eindruck auf mich. (1) |  |  |  |  |  |  |  |
| Dr. Müller überzeugt mich. (2) |  |  |  |  |  |  |  |
| Ich würde Dr. Müller vertrauen. (3) |  |  |  |  |  |  |  |

Q8.7


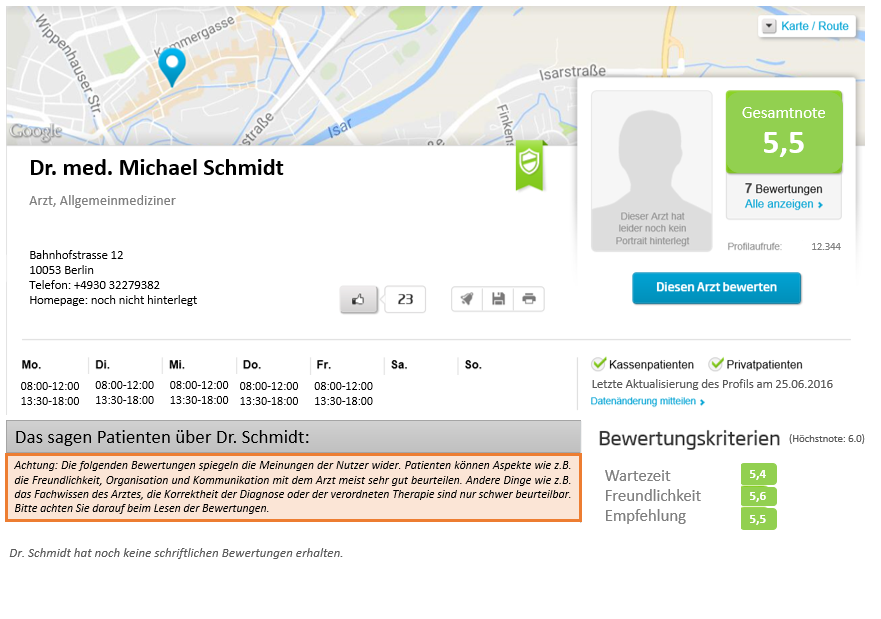


Q8.8 Bitte beurteilen Sie das Profil von Dr. Schmidt:

|  | 1 (1) | 2 (2) | 3 (3) | 4 (4) | 5 (5) |
| --- | --- | --- | --- | --- | --- |
| Hilfreich:Nutzlos (Q14.1_1) |  |  |  |  |  |
| Informativ:Verwirrend (Q14.1_2) |  |  |  |  |  |
| Glaubwürdig:Unglaubwürdig (Q14.1_3) |  |  |  |  |  |
| Vertrauenserweckend:Irreführend (Q14.1_4) |  |  |  |  |  |
| Verlässlich:Unzuverlässig (Q14.1_5) |  |  |  |  |  |
| Langweilig:Interessant (Q14.1_6) |  |  |  |  |  |
| Übersichtlich:Ungeordnet (Q14.1_7) |  |  |  |  |  |
| Realistisch:Verfälscht (Q14.1_8) |  |  |  |  |  |

Q8.9 Bitte geben Sie an, wie Sie Dr. Schmidt nach dem Lesen der online-Bewertung beurteilen:

|  | 1 (1) | 2 (2) | 3 (3) | 4 (4) | 5 (5) | 6 (6) | 7 (7) |
| --- | --- | --- | --- | --- | --- | --- | --- |
| Dr. Schmidt macht einen guten Eindruck auf mich. (1) |  |  |  |  |  |  |  |
| Dr. Schmidt überzeugt mich. (2) |  |  |  |  |  |  |  |
| Ich würde Dr. Schmdit vertrauen. (3) |  |  |  |  |  |  |  |

Q9.1


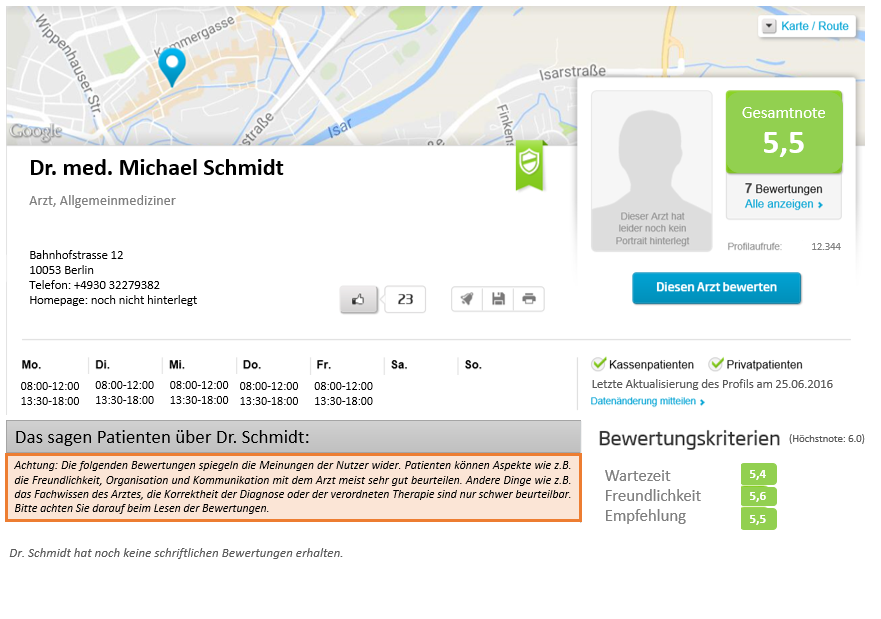


Q9.2


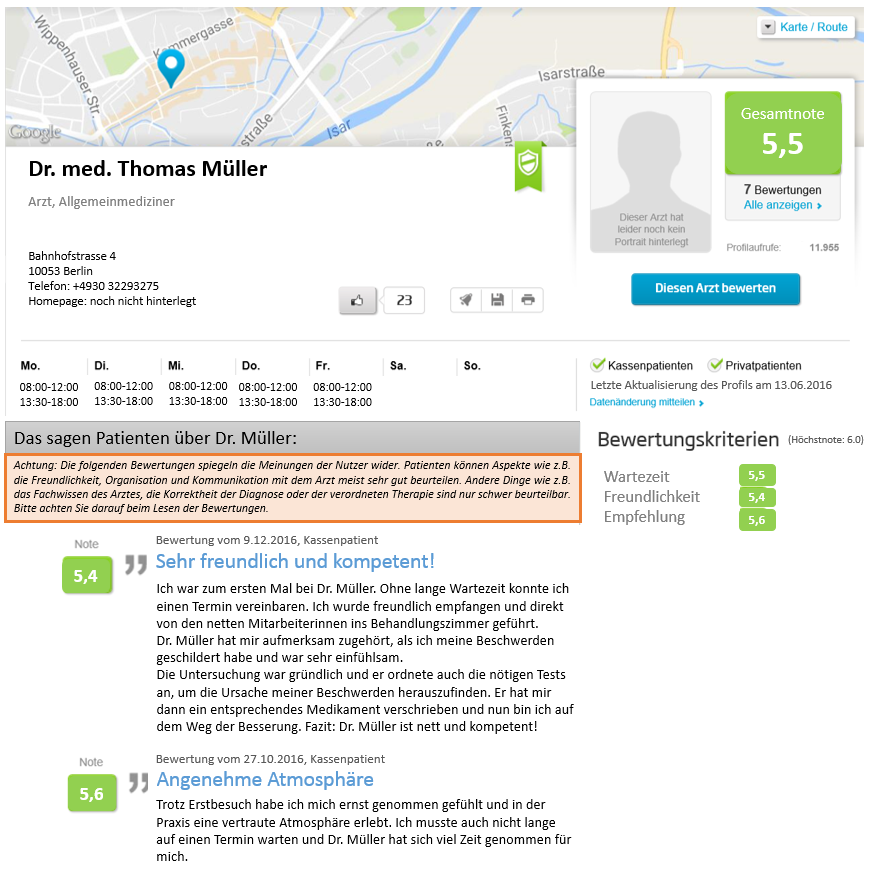


Q9.3 Nachdem Sie die beiden Profile der Ärzte durchgelesen haben, welchen Arzt würden Sie konsultieren?

- Definitiv Dr. Müller (1)
- Wahrscheinlich Dr. Müller (2)
- Eher Dr. Müller (3)
- Weder Dr. Müller noch Dr. Schmidt (4)
- Eher Dr. Schmidt (5)
- Wahrscheinlich Dr. Schmidt (6)
- Definitiv Dr. Schmidt (7)

Q9.4


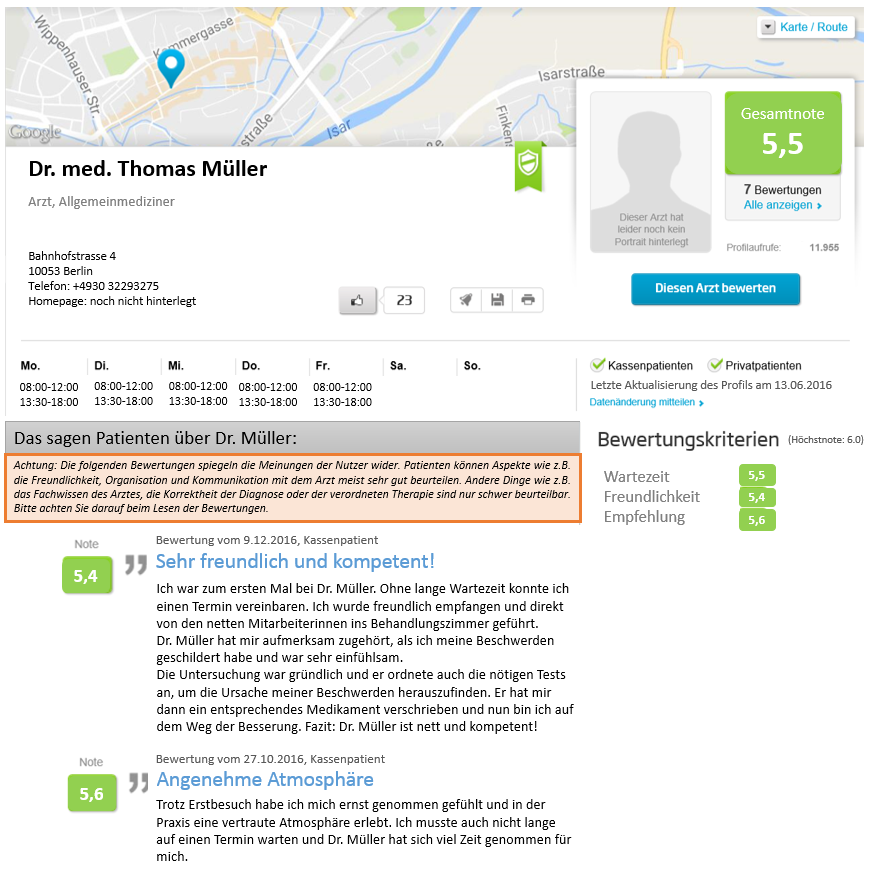


Q9.5 Bitte beurteilen Sie das Profil von Dr. Müller:

|  | 1 (1) | 2 (2) | 3 (3) | 4 (4) | 5 (5) |
| --- | --- | --- | --- | --- | --- |
| Hilfreich:Nutzlos (Q14.1_1) |  |  |  |  |  |
| Informativ:Verwirrend (Q14.1_2) |  |  |  |  |  |
| Glaubwürdig:Unglaubwürdig (Q14.1_3) |  |  |  |  |  |
| Vertrauenserweckend:Irreführend (Q14.1_4) |  |  |  |  |  |
| Verlässlich:Unzuverlässig (Q14.1_5) |  |  |  |  |  |
| Langweilig:Interessant (Q14.1_6) |  |  |  |  |  |
| Übersichtlich:Ungeordnet (Q14.1_7) |  |  |  |  |  |
| Realistisch:Verfälscht (Q14.1_8) |  |  |  |  |  |

Q9.6 Bitte geben Sie an, wie Sie Dr. Müller nach dem Lesen der online-Bewertung, beurteilen:

|  | 1 (1) | 2 (2) | 3 (3) | 4 (4) | 5 (5) | 6 (6) | 7 (7) |
| --- | --- | --- | --- | --- | --- | --- | --- |
| Dr. Müller macht einen guten Eindruck auf mich. (1) |  |  |  |  |  |  |  |
| Dr. Müller überzeugt mich. (2) |  |  |  |  |  |  |  |
| Ich würde Dr. Müller vertrauen. (3) |  |  |  |  |  |  |  |

Q9.7


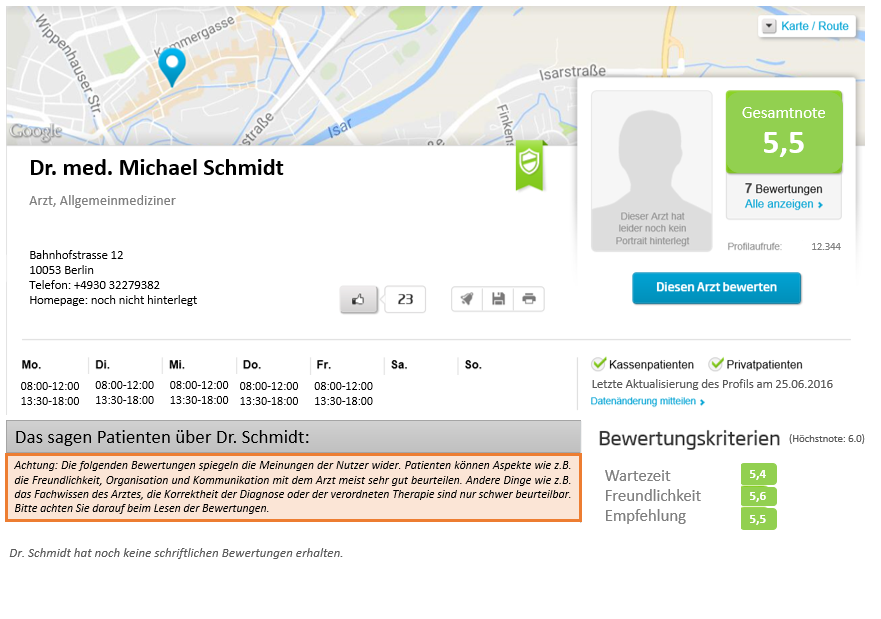


Q9.9 Bitte beurteilen Sie das Profil von Dr. Schmidt:

|  | 1 (1) | 2 (2) | 3 (3) | 4 (4) | 5 (5) |
| --- | --- | --- | --- | --- | --- |
| Hilfreich:Nutzlos (Q14.1_1) |  |  |  |  |  |
| Informativ:Verwirrend (Q14.1_2) |  |  |  |  |  |
| Glaubwürdig:Unglaubwürdig (Q14.1_3) |  |  |  |  |  |
| Vertrauenserweckend:Irreführend (Q14.1_4) |  |  |  |  |  |
| Verlässlich:Unzuverlässig (Q14.1_5) |  |  |  |  |  |
| Langweilig:Interessant (Q14.1_6) |  |  |  |  |  |
| Übersichtlich:Ungeordnet (Q14.1_7) |  |  |  |  |  |
| Realistisch:Verfälscht (Q14.1_8) |  |  |  |  |  |

Q9.8 Bitte geben Sie an, wie Sie Dr. Schmidt nach dem Lesen der online-Bewertung beurteilen:

|  | 1 (1) | 2 (2) | 3 (3) | 4 (4) | 5 (5) | 6 (6) | 7 (7) |
| --- | --- | --- | --- | --- | --- | --- | --- |
| Dr. Schmidt macht einen guten Eindruck auf mich. (1) |  |  |  |  |  |  |  |
| Dr. Schmidt überzeugt mich. (2) |  |  |  |  |  |  |  |
| Ich würde Dr. Schmidt vertrauen. (3) |  |  |  |  |  |  |  |

Q10.1


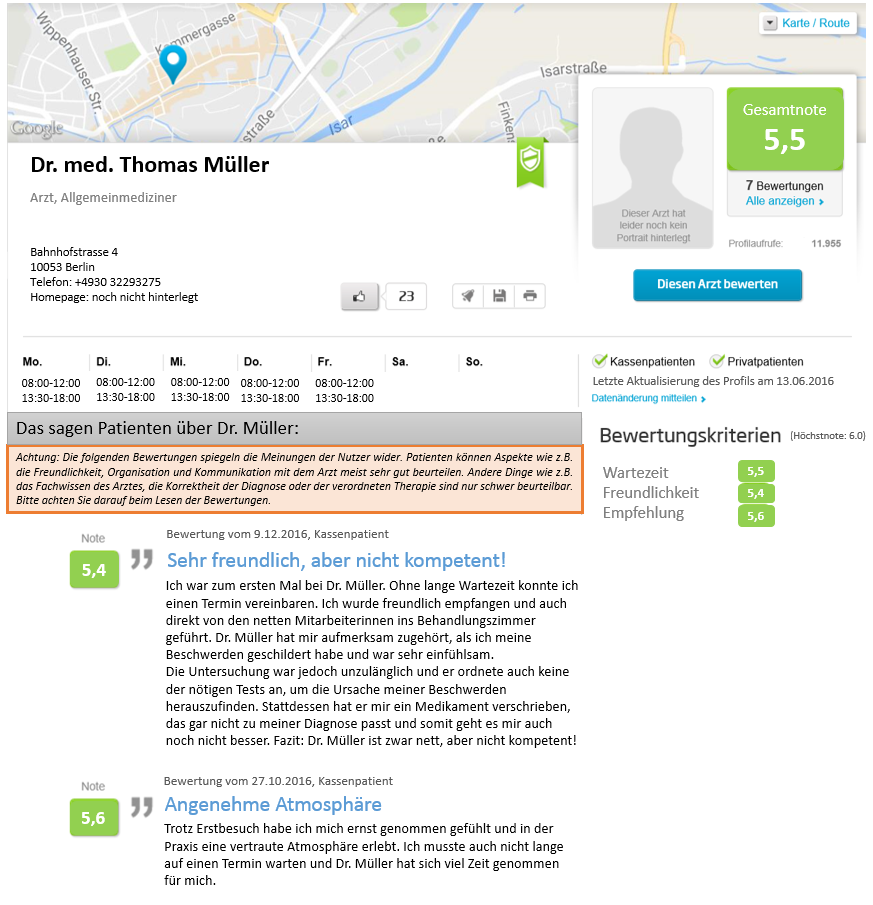


Q10.2


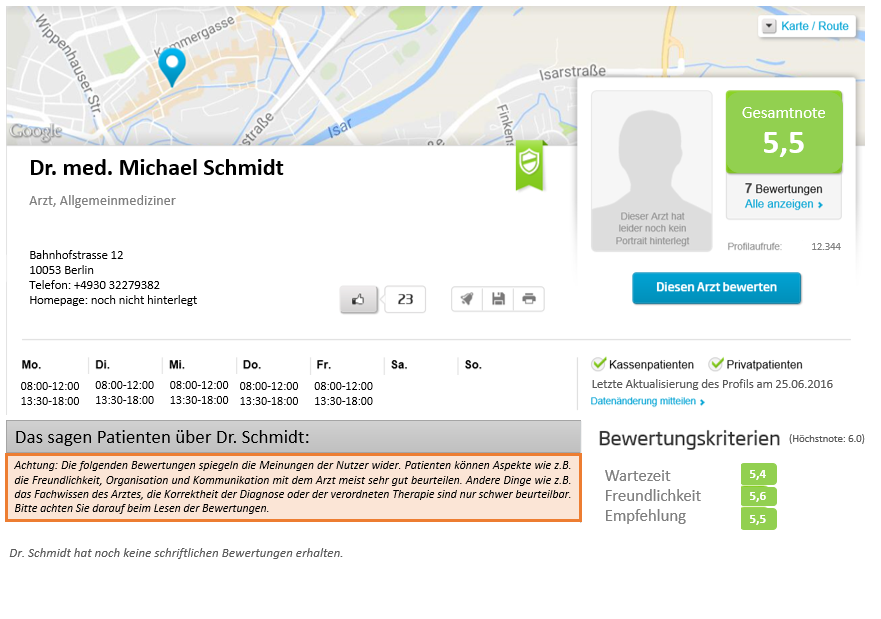


Q10.3 Nachdem Sie die beiden Profile der Ärzte durchgelesen haben, welchen Arzt würden Sie konsultieren?

- Definitiv Dr. Müller (1)
- Wahrscheinlich Dr. Müller (2)
- Eher Dr. Müller (3)
- Weder Dr. Müller noch Dr. Schmidt (4)
- Eher Dr. Schmidt (5)
- Wahrscheinlich Dr. Schmidt (6)
- Definitiv Dr. Schmidt (7)

Q10.4


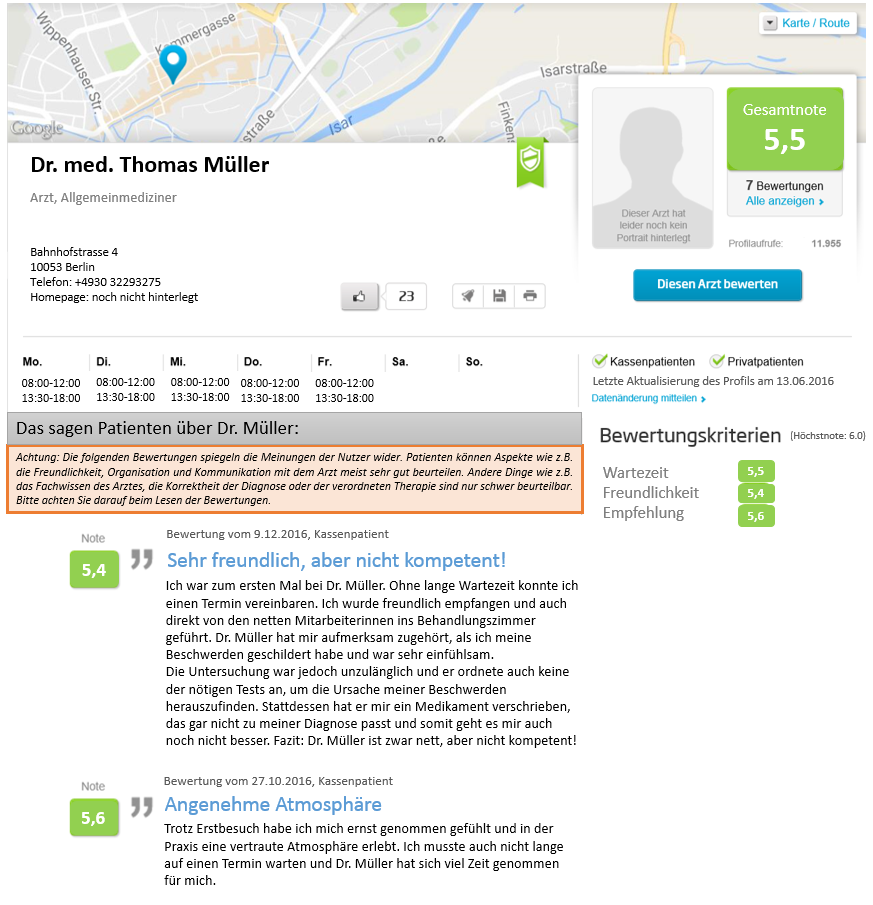


Q10.5 Bitte beurteilen Sie das Profil von Dr. Müller:

|  | 1 (1) | 2 (2) | 3 (3) | 4 (4) | 5 (5) |
| --- | --- | --- | --- | --- | --- |
| Hilfreich:Nutzlos (Q14.1_1) |  |  |  |  |  |
| Informativ:Verwirrend (Q14.1_2) |  |  |  |  |  |
| Glaubwürdig:Unglaubwürdig (Q14.1_3) |  |  |  |  |  |
| Vertrauenserweckend:Irreführend (Q14.1_4) |  |  |  |  |  |
| Verlässlich:Unzuverlässig (Q14.1_5) |  |  |  |  |  |
| Langweilig:Interessant (Q14.1_6) |  |  |  |  |  |
| Übersichtlich:Ungeordnet (Q14.1_7) |  |  |  |  |  |
| Realistisch:Verfälscht (Q14.1_8) |  |  |  |  |  |

Q10.6 Bitte geben Sie an, wie Sie Dr. Müller nach dem Lesen der online-Bewertung, beurteilen:

|  | 1 (1) | 2 (2) | 3 (3) | 4 (4) | 5 (5) | 6 (6) | 7 (7) |
| --- | --- | --- | --- | --- | --- | --- | --- |
| Dr. Müller macht einen guten Eindruck auf mich. (1) |  |  |  |  |  |  |  |
| Dr. Müller überzeugt mich. (2) |  |  |  |  |  |  |  |
| Ich würde Dr. Müller vertrauen. (3) |  |  |  |  |  |  |  |

Q10.7


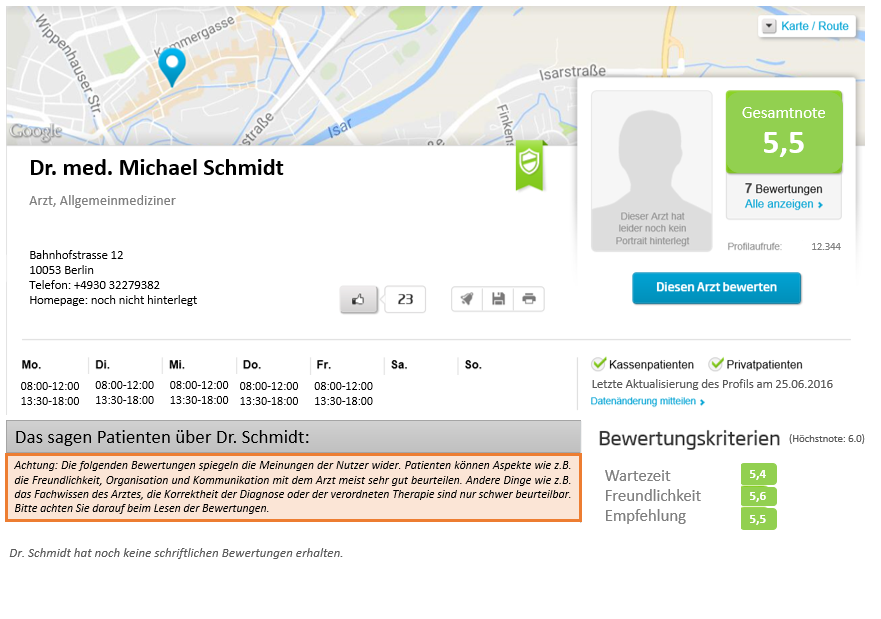


Q10.9 Bitte beurteilen Sie das Profil von Dr. Schmidt:

|  | 1 (1) | 2 (2) | 3 (3) | 4 (4) | 5 (5) |
| --- | --- | --- | --- | --- | --- |
| Hilfreich:Nutzlos (Q14.1_1) |  |  |  |  |  |
| Informativ:Verwirrend (Q14.1_2) |  |  |  |  |  |
| Glaubwürdig:Unglaubwürdig (Q14.1_3) |  |  |  |  |  |
| Vertrauenserweckend:Irreführend (Q14.1_4) |  |  |  |  |  |
| Verlässlich:Unzuverlässig (Q14.1_5) |  |  |  |  |  |
| Langweilig:Interessant (Q14.1_6) |  |  |  |  |  |
| Übersichtlich:Ungeordnet (Q14.1_7) |  |  |  |  |  |
| Realistisch:Verfälscht (Q14.1_8) |  |  |  |  |  |

Q10.8 Bitte geben Sie an, wie Sie Dr. Schmidt nach dem Lesen der online-Bewertung beurteilen:

|  | 1 (1) | 2 (2) | 3 (3) | 4 (4) | 5 (5) | 6 (6) | 7 (7) |
| --- | --- | --- | --- | --- | --- | --- | --- |
| Dr. Schmidt macht einen guten Eindruck auf mich. (1) |  |  |  |  |  |  |  |
| Dr. Schmidt überzeugt mich. (2) |  |  |  |  |  |  |  |
| Ich würde Dr. Schmidt vertrauen. (3) |  |  |  |  |  |  |  |

Q11.1


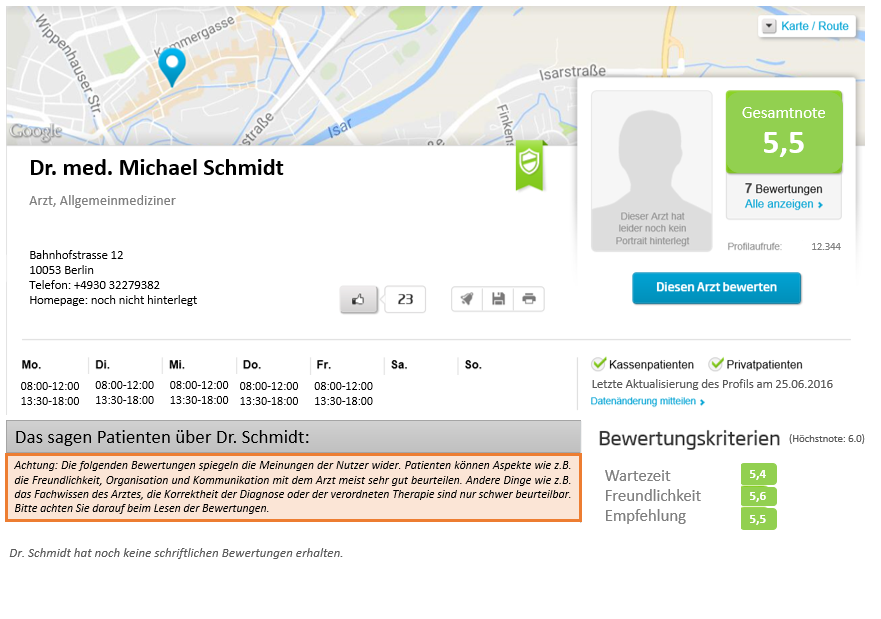


Q11.2


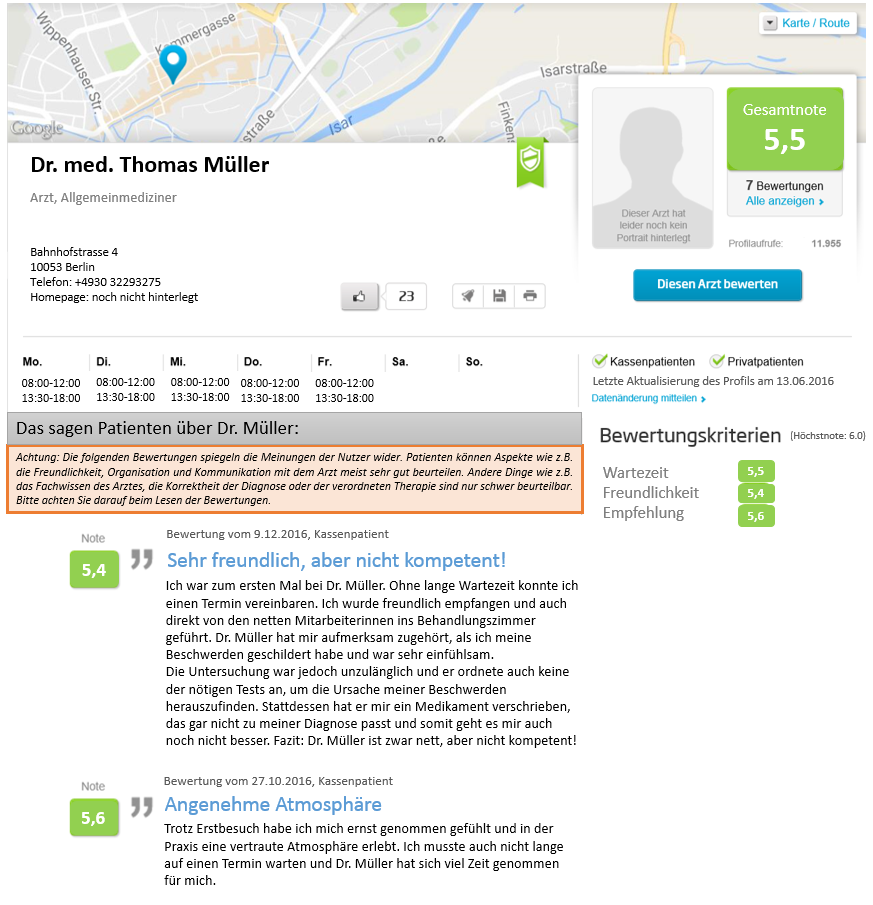


Q11.3 Nachdem Sie die beiden Profile der Ärzte durchgelesen haben, welchen Arzt würden Sie konsultieren?

- Definitiv Dr. Müller (1)
- Wahrscheinlich Dr. Müller (2)
- Eher Dr. Müller (3)
- Weder Dr. Müller noch Dr. Schmidt (4)
- Eher Dr. Schmidt (5)
- Wahrscheinlich Dr. Schmidt (6)
- Definitiv Dr. Schmidt (7)

Q11.4


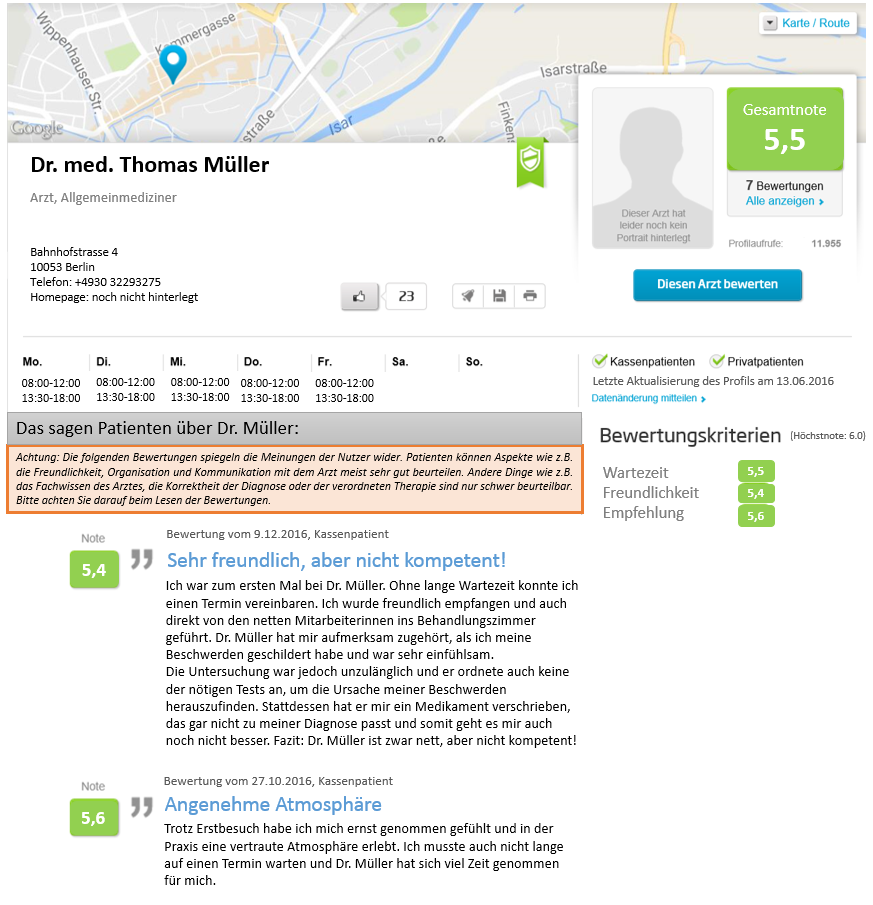


Q11.5 Bitte beurteilen Sie das Profil von Dr. Müller:

|  | 1 (1) | 2 (2) | 3 (3) | 4 (4) | 5 (5) |
| --- | --- | --- | --- | --- | --- |
| Hilfreich:Nutzlos (Q14.1_1) |  |  |  |  |  |
| Informativ:Verwirrend (Q14.1_2) |  |  |  |  |  |
| Glaubwürdig:Unglaubwürdig (Q14.1_3) |  |  |  |  |  |
| Vertrauenserweckend:Irreführend (Q14.1_4) |  |  |  |  |  |
| Verlässlich:Unzuverlässig (Q14.1_5) |  |  |  |  |  |
| Langweilig:Interessant (Q14.1_6) |  |  |  |  |  |
| Übersichtlich:Ungeordnet (Q14.1_7) |  |  |  |  |  |
| Realistisch:Verfälscht (Q14.1_8) |  |  |  |  |  |

Q11.6 Bitte geben Sie an, wie Sie Dr. Müller nach dem Lesen der online-Bewertung, beurteilen:

|  | 1 (1) | 2 (2) | 3 (3) | 4 (4) | 5 (5) | 6 (6) | 7 (7) |
| --- | --- | --- | --- | --- | --- | --- | --- |
| Dr. Müller macht einen guten Eindruck auf mich. (1) |  |  |  |  |  |  |  |
| Dr. Müller überzeugt mich. (2) |  |  |  |  |  |  |  |
| Ich würde Dr. Müller vertrauen. (3) |  |  |  |  |  |  |  |

Q11.7


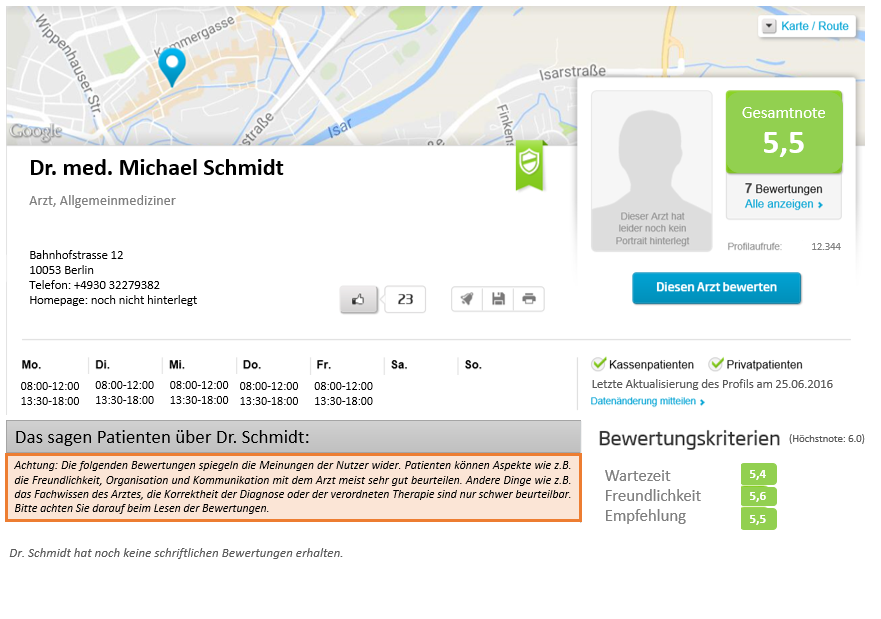


Q11.9 Bitte beurteilen Sie das Profil von Dr. Schmidt:

|  | 1 (1) | 2 (2) | 3 (3) | 4 (4) | 5 (5) |
| --- | --- | --- | --- | --- | --- |
| Hilfreich:Nutzlos (Q14.1_1) |  |  |  |  |  |
| Informativ:Verwirrend (Q14.1_2) |  |  |  |  |  |
| Glaubwürdig:Unglaubwürdig (Q14.1_3) |  |  |  |  |  |
| Vertrauenserweckend:Irreführend (Q14.1_4) |  |  |  |  |  |
| Verlässlich:Unzuverlässig (Q14.1_5) |  |  |  |  |  |
| Langweilig:Interessant (Q14.1_6) |  |  |  |  |  |
| Übersichtlich:Ungeordnet (Q14.1_7) |  |  |  |  |  |
| Realistisch:Verfälscht (Q14.1_8) |  |  |  |  |  |

Q11.8 Bitte geben Sie an, wie Sie Dr. Schmidt nach dem Lesen der online-Bewertung beurteilen:

|  | 1 (1) | 2 (2) | 3 (3) | 4 (4) | 5 (5) | 6 (6) | 7 (7) |
| --- | --- | --- | --- | --- | --- | --- | --- |
| Dr. Schmidt macht einen guten Eindruck auf mich. (1) |  |  |  |  |  |  |  |
| Dr. Schmidt überzeugt mich. (2) |  |  |  |  |  |  |  |
| Ich würde Dr. Schmidt vertrauen. (3) |  |  |  |  |  |  |  |

Q12.1 Nun bitten wir Sie noch einige Fragen zu Ihrer allgemeinen Einschätzung der beiden Arztprofile zu beantworten: Würden sie sich sicher fühlen, eine Entscheidung zu treffen, basierend auf den Informationen, die Sie gesehen haben?

- 1 = Nein, ich würde mich gar nicht sicher fühlen (1)
- 2 (2)
- 3 (3)
- 4 (4)
- 5 (5)
- 6 (6)
- 7 = Ja, ich würde mich sehr sicher fühlen (7)

Q12.2 Würden Sie noch eine weitere Arztbewertungswebseite konsultieren, um mehr Informationen zu erhalten bevor Sie eine Entscheidung treffen würden?

- 1 = Nein, sicher nicht (1)
- 2 (2)
- 3 (3)
- 4 (4)
- 5 (5)
- 6 (6)
- 7 = Ja, ganz sicher (7)

Q12.3 Wie vertrauenswürdig waren die verfassten Arztbewertungen Ihrer Meinung nach?

- 1 = Überhaupt nicht vertrauenswürdig (1)
- 2 (2)
- 3 (3)
- 4 (4)
- 5 (5)
- 6 (6)
- 7 = Sehr vertrauenswürdig (7)

Q12.4 Überlegen Sie bitte, ob folgende Aussagen auf Sie persönlich zutreffen oder nicht:

|  | 1 (1) | 2 (2) | 3 (3) | 4 (4) | 5 (5) | 6 (6) | 7 (7) |
| --- | --- | --- | --- | --- | --- | --- | --- |
| Meiner Meinung nach ist es lohnenswert, sich online-Bewertungen anderer Nutzer anzuschauen. (1) |  |  |  |  |  |  |  |
| Ich glaube, es ist gut für mich, online-Bewertungen anderer Nutzer durchzulesen. (2) |  |  |  |  |  |  |  |
| Alles in allem ist meine Einstellung gegenüber online-Bewertungen anderer Nutzer positiv. (3) |  |  |  |  |  |  |  |

Q13.1 Bitte kreuzen Sie an, ob Sie den folgenden Aussagen zustimmen oder nicht:

|  | 1 (1) | 2 (2) | 3 (3) | 4 (4) | 5 (5) |
| --- | --- | --- | --- | --- | --- |
| Ich kann mich kaum darauf verlassen, dass Bewertungen von Ärzten der Wahrheit entsprechen. (Q13.1_1 eWOM_Bewertungen von Aerzten entsprechen der Wahrheit) |  |  |  |  |  |
| Online-Bewertungen von Ärzten sind grundsätzlich nicht wahrheitsgetreu. (Q13.1_2 eWOM_online-Bewertungen von Aerzten sind nicht wahrheitsgetreu) |  |  |  |  |  |
| Im Allgemeinen geben Bewertungen im Internet nicht ein wahres Bild davon ab, wie ein Arzt wirklich ist. (Q13.1_3_eWOM_Bewertungen geben nicht ien wahres Bild davon ab, wie ein Arzt wirklich ist) |  |  |  |  |  |
| Patienten, die online-Bewertungen schreiben, beabsichtigen immer etwas damit. (Q13.1_7_eWOM_online-Bewertungen wollen in die Irre fuehren) |  |  |  |  |  |
| Patienten, die negative Bewertungen über einen Arzt schreiben, wollen sich rächen. (Q13.1_4_eWOM negative Bewertungen wollen sich raechen) |  |  |  |  |  |
| Patienten können nicht wirklich einschätzen wie gut ein Arzt ist und sollten Ärzte deshalb auch nicht im Internet bewerten. (Q13.1_5_eWOM_Patienten koennen Arzt nicht bewerten) |  |  |  |  |  |
| Die meisten Arztbewertungen beabsichtigen einen in die Irre zu führen. (Q13.1_6_eWOM_Arztbewertungen wollen in die Irre fuehren) |  |  |  |  |  |
| Leute, die Arztbewertungen verfassen, sind nicht unbedingt echte Patienten dieses Arztes. (Q13.1_8_eWOM_Verfasser von Bewertungen sind nicht echte Patienten) |  |  |  |  |  |
| Leute, die online-Bewertungen schreiben, geben vor, jemand anderes zu sein. (Q13.1_9_eWOM_online-Bewerter geben vor jemand anderes zu sein) |  |  |  |  |  |
| Verschiedene Bewertungen sind oft von den selben Leuten veröffentlicht, jedoch unter unterschiedlichen Namen. (Q13.1_10_eWOM_Bewertungen sind von den selben Leuten unter unterschiedlichen Namen) |  |  |  |  |  |

Q14.1 Bitte beantworten Sie die unten stehenden Fragen OHNE einen Taschenrechner zu benutzen. Gerne dürfen Sie sich aber handschriftliche Notizen machen.

Q14.2 Stellen Sie sich vor, wir werfen ein normale Münze 1000 mal. Einmal angenommen, auf einer Seite ist ein Kopf abgebildet: Wie viele Male, denken Sie, wird die Münze mit dem Kopf oben landen?

So viele Male von 1000 Würfen: (1)

Q14.3 Bei einer Bingo-Lotterie liegt die Chance, CHF10 zu gewinnen, bei 1 %. Was schätzen Sie: Wie viele von 1000 Leuten werden diese CHF10 gewinnen, wenn jeder ein Einzel-Ticket für die Bingo-Lotterie kauft?

So viele von 1000 Person(en): (1)

Q14.4 Bei einer Verlosung ist die Chance, ein Auto zu gewinnen, 1 zu 1000. Wieviel Prozent der Lose gewinnen ein Auto?

So viel Prozent (% ) der Lose: (1)

Q15.1 Bitte lesen Sie das unten aufgeführte Etikett durch. Es stammt von einer Glacépackung. Bitte beantworten Sie anschliessend die Fragen, die sich darauf beziehen.

Q15.2 Nährwertdeklaration:

| Portionen pro Packung: 4 | | |
| --- | --- | --- |
| Pro Portion | % GDA* | |
| Nährwerte Ø | 250 kcal. | 20% |
| Fett | 13 gr. | 40% |
| davon gesättigte Fettsäuren | 9 gr. | 12% |
| Cholesterol | 28 mg. | 2% |
| Natrium | 55 mg. | 12% |
| Kohlenhydrate | 30 gr. | |
| davon Zucker            davon Ballaststoffe | 23 gr. 2 gr. | |
| Eiweiss | 4 gr. | 8% |
| *Richtwert für die Tageszufuhr basierend auf einer ausgewogenen Ernährung eines durchschnittlichen Erwachsenen von täglich 2,000 kcal. Der Nährstoffbedarf variiert je nach Alter, Geschlecht, körperlicher Aktivität, etc. | | |
| Zutaten: Rahm, Magermilch, verflüssigter Zucker, Wasser, Eigelb, brauner Zucker, Milchfett, Erdnussöl, Zucker, Butter, Salz, Carr | | |

Q15.3 Wenn Sie die ganze Glacépackung essen, wie viele Kalorien würden Sie zu sich nehmen?

Anzahl Kalorien: ____________

Q15.4 Wenn Sie als Zwischenmahlzeit 60g Kohlenhydrate essen dürfen, wie viele Portionen Glacé könnten Sie essen?

Anzahl Portion(en): ____________

Q15.5 Ihr Arzt rät Ihnen, in Ihrem Ernährungsplan die Menge der gesättigten Fettsäuren zu reduzieren. Normalerweise nehmen Sie täglich insgesamt 42g gesättigte Fettsäuren zu sich, was pro Tag auch eine Portion Glacé beinhaltet. Wenn Sie damit aufhören würden Glacé zu essen, wie viel Gramm gesättigte Fettsäuren würden Sie täglich konsumieren?

Anzahl Gramm (g): ____________

Q15.6 Täglich konsumieren Sie normalerweise 2500 Kalorien. Wie viel Prozent Ihres täglichen Kalorienbedarfs würden Sie zu sich nehmen, wenn Sie eine Portion Glacé essen?

So viel Prozent (%):____________

Q15.7 Stellen Sie sich vor, Sie wären auf folgende Dinge allergisch: Penicillin, Erdnüsse, Latexhandschuhe und Bienenstiche. Wäre es sicher für Sie, diese Glacé zu essen?

- Ja (1)
- Nein (2)
- Weiss nicht. (3)

Display This Question:

If Stellen Sie sich vor, Sie wären auf folgende Dinge allergisch: Penicillin, Erdnüsse, Latexhandsch... Nein Is Selected

Q15.8 Warum wäre es nicht sicher? (Bitte Antwort unten eintragen)

____________________________________

Q16.1 Bitte kreuzen Sie an, wie stark Sie den folgenden Aussagen zum Umgang mit Gesundheitsinformationen aus dem Internet zustimmen.

|  | 0 (1) | 1 (2) | 2 (3) | 3 (4) | 4 (5) |
| --- | --- | --- | --- | --- | --- |
| Ich weiss, wie ich im Internet nützliche Gesundheitsinformationen finde. (1) |  |  |  |  |  |
| Ich weiss, wie ich das Internet nutzen kann, um Antworten auf meine Fragen rund um das Thema Gesundheit zu bekommen. (2) |  |  |  |  |  |
| Ich weiss, wo im Internet ich nützliche Gesundheitsinformationen finden kann. (3) |  |  |  |  |  |
| Ich weiss, wie ich Informationen aus dem Internet so nutzen kann, dass sie mir weiterhelfen. (4) |  |  |  |  |  |
| Ich bin in der Lage, Informationen, die ich im Internet finde, kritisch zu bewerten. (5) |  |  |  |  |  |
| Ich kann im Internet zuverlässige von fragwürdigen Informationen unterscheiden. (6) |  |  |  |  |  |
| Wenn ich gesundheitsbezogene Entscheidungen auf Basis von Informationen aus dem Internet treffe, fühle ich mich dabei sicher. (7) |  |  |  |  |  |

Q17.1 Stellen Sie sich vor, Sie wären zum ersten Mal bei einem neuen Arzt gewesen und man würde sie nun darum bitten, eine Bewertung abzugeben.

Wie gut könnten Sie die unten beschriebenen Kategorien bewerten?

|  | 1 (1) | 2 (2) | 3 (3) | 4 (4) | 5 (5) | 6 (6) | 7 (7) |
| --- | --- | --- | --- | --- | --- | --- | --- |
| Organisation der Praxis (z.B. Terminvereinbarung- und Planung, Wartezeit in der Praxis, Benachrichtigung bei Verspätungen) (1) |  |  |  |  |  |  |  |
| Personal (z.B. Freundlichkeit, Hilfsbereitschaft) (2) |  |  |  |  |  |  |  |
| Technische/ medizinische Fähigkeiten des Arztes (z.B. Kompetenz, korrekte Diagnosestellung, Anordnung der richtigen Tests um zur Diagnose zu gelangen, Qualität der Behandlung) (3) |  |  |  |  |  |  |  |
| Zwischenmenschliche Fähigkeiten des Arztes (z.B. Qualität und Verständlichkeit der mitgeteilten Informationen, Empathie und Verhalten gegenüber dem Patienten, Fähigkeit zuzuhören) (4) |  |  |  |  |  |  |  |
| Behandlungsresultat (z.B. Wirkkraft der Behandlung, Verbesserung des Gesundheitszustands im erwarteten Ausmass, Kosten-Nutzen Verhältnis) (5) |  |  |  |  |  |  |  |

Q18.1 Bitte beantworten Sie zum Schluss noch einige Fragen zu Ihrer Person.

Q18.2 Wie alt sind Sie? ____________

Q18.3 Sind Sie männlich oder weiblich?

- Männlich (1)
- Weiblich (2)

Q18.4 Welches ist Ihr höchster Bildungsabschluss?

- Primarschule (1)
- Sekundar- oder Realschule (2)
- Berufslehre (3)
- Matura oder administrativer Abschluss (Berufsschule, Handelsschule, o.Ä.) (4)
- Fachhochschulabschluss (5)
- Universitätsabschluss (6)

Q18.5 Arbeiten Sie in einem medizinischen Beruf, in welchem Sie mit Patienten in Kontakt sind?

- Ja, ich bin Arzt/ Ärztin. (1)
- Ja, ich arbeite in einem medizinischen Beruf mit Patientenkontakt, bin aber nicht Arzt/ Ärztin. (2)
- Nein. (3)

Q19.1 Vielen Dank für Ihre Teilnahme an dieser Studie.

In diesem Experiment haben Sie eine von vier möglichen Arztbewertungswebseiten mit je zwei Arztprofilen gesehen.

Diese unterscheiden wie folgt:

1. Entweder sahen Sie positive oder negative schriftliche Bewertungen.

2. Entweder war ein Warnhinweis präsent oder nicht, der darauf aufmerksam machte, dass solche Bewertungen subjektiv sind.

Diese Studie untersucht, ob diese unterschiedlichen Webseiten/ Arztprofile die Meinung gegenüber den gezeigten Ärzten und Bewertungsportalen beeinflussen.

Um die Studie zu beenden, klicken Sie bitte unten auf "weiter" (roter Knopf).

---

Vielen Dank für Ihre Teilnahme. Ihre Antwort wurde erfasst und Sie haben den Fragebogen erfolgreich abgeschlossen. Wir haben Sie gebeten, einige Textverständnis- und Mathematikaufgaben zu lösen. Falls Sie an den korrekten Antworten interessiert sind, können Sie diese unten einsehen. Andernfalls können Sie den Browser nun schliessen.

Falls Sie noch Fragen zur Studie haben, wenden Sie sich bitte an:

Fabia Rothenfluh, M.Sc. (Doktorandin)
Prof. Dr. Peter J. Schulz (Projektleiter)
Università della Svizzera italiana
CH-6900 Lugano
Email: fabia.rothenfluh@usi.ch


**Korrekte Antworten zu den Text- und Mathematikaufgaben**:
 
1. Stellen Sie sich vor, wir werfen ein normale Münze 1000 mal. Einmal angenommen, auf einer Seite ist ein Kopf abgebildet: Wie viele Male, denken Sie, wird die Münze mit dem Kopf oben landen?

Korrekte Antwort: 500 Mal von 1000 Würfen  (≥ 400 und ≥ 600)

2. Bei einer Bingo-Lotterie liegt die Chance, CHF10 zu gewinnen, bei 1 %. Was schätzen Sie: Wie viele von 1000 Leuten werden diese CHF10 gewinnen, wenn jeder ein Einzel-Ticket für die Bingo-Lotterie kauft?

Korrekte Antwort: 10 Personen von 1000 

3. Bei einer Verlosung ist die Chance, ein Auto zu gewinnen, 1 zu 1000. Wieviel Prozent der Lose gewinnen ein Auto?

Korrekte Antwort: 0.1 % der Lose
 

-------------------------------------------------------------------------------
-------------------------------------------------------------------------------
 

Nährwertdeklaration:

| Portionen pro Packung: 4 | | |
| --- | --- | --- |
| Pro Portion | % GDA* | |
| Nährwerte Ø | 250 kcal. | 20% |
| Fett | 13 gr. | 40% |
| davon gesättigte Fettsäuren | 9 gr. | 12% |
| Cholesterol | 28 mg. | 2% |
| Natrium | 55 mg. | 12% |
| Kohlenhydrate | 30 gr. | |
| davon Zucker            davon Ballaststoffe | 23 gr. 2 gr. | |
| Eiweiss | 4 gr. | 8% |
| *Richtwert für die Tageszufuhr basierend auf einer ausgewogenen Ernährung eines durchschnittlichen Erwachsenen von täglich 2,000 kcal. Der Nährstoffbedarf variiert je nach Alter, Geschlecht, körperlicher Aktivität, etc. | | |
| Zutaten: Rahm, Magermilch, verflüssigter Zucker, Wasser, Eigelb, brauner Zucker, Milchfett, Erdnussöl, Zucker, Butter, Salz, Carrageen, Vanille-Extrakt. | | |

1. Wenn Sie das ganze Glacépackung essen, wie viele Kalorien würden Sie zu sich nehmen?

Korrekte Antwort: 1000 Kalorien

2. Wenn Sie als Zwischenmahlzeit 60g Kohlenhydrate essen dürfen, wie viel Glacé könnten Sie essen?

Korrekte Antwort: 2 Portionen

3. Ihr Arzt rät Ihnen in Ihrem Ernährungsplan die Menge der gesättigten Fettsäuren zu reduzieren. Normalerweise nehmen Sie täglich insgesamt 42g gesättigte Fettsäuren zu sich, was pro Tag auch eine Portion Glacé beinhaltet. Wenn Sie damit aufhören würden Glacé zu essen, wie viel Gramm gesättigte Fettsäuren würden Sie täglich konsumieren?

Korrekte Antwort: 33g

4. Wenn Sie  täglich normalerweise 2500 Kalorien zu sich nehmen, wie viel Prozent Ihres täglichen Kalorienbedarfs würden Sie zu sich nehmen, wenn Sie eine Portion essen?

Korrekte Antwort: 10%

Stellen Sie sich vor, Sie wären auf folgende Dinge allergisch: Penicillin, Erdnüsse, Latexhandschuhe und Bienenstiche.
 
5. Wäre es sicher für Sie, diese Glacé zu essen?

Korrekte Antwort: Nein.

6. Warum wäre es nicht sicher?

Korrekte Antwort: Die Glacé enthält Erdnussöl und da Sie auf Erdnüsse allergisch sind, wäre es nicht sicher für Sie diese Glacé zu essen.
